# Exploring the relationship between supervisory support, self-efficacy, and satisfaction among nursing students in Saudi Arabia

**DOI:** 10.1371/journal.pone.0352318

**Published:** 2026-07-16

**Authors:** Thurayya Eid, Faihan F. Alshaibany, Abdulaziz M. Alodhailah, Waleed M. Alshehri

**Affiliations:** 1 Department of Medical-Surgical Nursing, College of Nursing, King Saud University, Riyadh, Saudi Arabia; 2 Department of Nursing Administration and Education, College of Nursing, King Saud University, Riyadh, Saudi Arabia; University of Hafr Al-Batin, SAUDI ARABIA

## Abstract

**Background:**

Supervisory support during clinical placements is central to nursing students’ professional development. Professional self-efficacy, students’ confidence in performing clinical tasks, is increasingly recognised as a key outcome associated with transition to practice and workforce retention. In Saudi Arabia, Vision 2030 healthcare reforms have intensified demand for qualified nurses, making the quality of clinical supervision a strategic priority. This study examined the relationships among supervisory support, professional self-efficacy, and nursing student satisfaction at King Saud University-affiliated hospitals.

**Methods:**

This descriptive cross-sectional study was conducted among 145 nursing students (second-, third-, and fourth-year bachelor’s degree students) at three King Saud University-affiliated teaching hospitals in Riyadh, Saudi Arabia. Participants were selected using stratified random sampling based on year of study. Data were collected using four validated instruments: a sociodemographic questionnaire, the Supervisory Support Scale (9 items; Cronbach’s α = 0.90), the Nursing Student Satisfaction Scale (6 items; α = 0.88), and an adapted Professional Self-Efficacy Scale (12 items; α = 0.90). Descriptive statistics summarised the three key study variables (supervisory support, professional self-efficacy, and student satisfaction) alongside sociodemographic characteristics. Pearson correlation coefficients assessed the strength and direction of linear associations among continuous study variables. Formal mediation analysis was conducted using the PROCESS macro (Model 4) with 5,000 bias-corrected bootstrap resampling iterations.

**Results:**

Of 175 students approached and meeting eligibility criteria, 145 returned valid questionnaires (response rate = 82.9%). The sample was predominantly female (60.7%) and in the age range of 20–22 years (66.9%). Students reported high levels of supervisory support (mean = 4.10 ± 0.53 out of 5), satisfaction with clinical placements (mean = 4.11 ± 0.52), and professional self-efficacy (mean = 4.06 ± 0.56). Supervisory support was strongly correlated with satisfaction (r = 0.68, p < 0.001) and professional self-efficacy (r = 0.57, p < 0.001); professional self-efficacy was positively correlated with satisfaction (r = 0.49, p < 0.001Statistical mediation analysis revealed that professional self-efficacy partially mediated the supervisory support–satisfaction relationship: the indirect association (ab = 0.13, 95% CI [0.07, 0.19]) was statistically significant and accounted for approximately 19% of the total association. In regression analysis, supervisory support remained significantly associated with satisfaction (β = 0.43, p < 0.001) even after accounting for professional self-efficacy, consistent with partial statistical mediation.

**Conclusions:**

Supervisory support was significantly and positively associated with nursing students’ satisfaction and professional self-efficacy. These findings, grounded in social cognitive theory, suggest that high-quality supervision may enhance clinical learning outcomes both directly and through strengthening students’ professional confidence. Standardising supervisory practices, investing in supervisor training, and creating structured feedback mechanisms are priorities for improving nursing education in Saudi Arabia and advancing national nursing workforce goals under Vision 2030.

## Background

Clinical experience serves as a crucial bridge between theoretical learning and practical application in nursing education [1]. During clinical placements, nursing students rely on clinical instructors, mentors, and staff nurses for supervision and guidance as they develop the skills needed for safe patient care [2]. Supervisory support significantly shapes the quality of this experience, influencing academic performance, professional growth, self-confidence, and satisfaction [3,4]. Effective supervision creates environments where students develop critical thinking, clinical decision-making, and hands-on nursing skills [3,5,6].

Despite broad consensus on the value of supervision, significant challenges persist. Clinical instructors often face high workloads and variable caseloads that limit individualised student contact [7,8]. Supervision quality varies considerably across sites owing to differences in instructor experience, institutional resources, and organisational culture [9,10]. Students frequently report receiving insufficient feedback and encountering communication gaps with supervisors, which can undermine confidence and learning outcomes [3,5]. In Saudi Arabia, these challenges are compounded by a hierarchical cultural framework that may shape how students engage with supervisors and the type of support they feel able to request [11].

In Saudi Arabia, the demand for trained nurses has grown alongside expanding healthcare services and the rise of chronic disease [12]. The Vision 2030 initiative has intensified this demand by calling for a qualified, locally trained nursing workforce capable of delivering high-quality care [13]. Nursing programmes have responded by strengthening clinical training in teaching hospitals, where supervisory support remains central to student learning [14]. Clinical instructors who guide, mentor, and evaluate students in real-world settings play a direct role in shaping clinical competence, professional identity, and readiness for independent practice [15,16,17].

Supervisory support in clinical education encompasses the guidance, mentorship, and structured feedback that students receive from instructors and experienced nurses during hospital placements [18]. International research consistently demonstrates that strong supervision is associated with higher student satisfaction, greater self-efficacy, and improved academic performance [19–21]. Conversely, students who experience weak or inconsistent supervision report greater stress, feelings of inadequacy, and lower satisfaction, all of which hinder professional development [22,23]. Well-supported students are more likely to transition smoothly into nursing practice, demonstrating greater clinical competence and confidence [24,25].

Professional self-efficacy, students’ confidence in performing clinical and professional tasks, is a particularly important outcome in this context. Higher self-efficacy is associated with better clinical performance, lower stress, and smoother transition to practice [26]. According to Bandura’s social cognitive theory, self-efficacy develops through mastery experiences, vicarious learning, verbal persuasion, and physiological states [27]. In the clinical education context, Bandura’s [27] four sources of self-efficacy are operationalised as follows: mastery experience, through supervised clinical tasks of progressively increasing complexity; vicarious learning, through observation of competent instructors and peers performing clinical skills; verbal persuasion, through constructive, specific feedback delivered by supervisors; and management of physiological states, through strategies that reduce anxiety during clinical performance [21,28]. Clinical supervisors occupy a central role in activating these pathways through feedback, role-modelling, and guided practice [29]. Yet empirical evidence on how supervisory support, self-efficacy, and satisfaction inter-relate among undergraduate nursing students in non-Western settings remains limited.

A notable gap in the existing literature is the lack of systematic research examining these relationships specifically in Saudi Arabia. Although some studies have explored broader aspects of the clinical learning environment, few have investigated how supervision and self-efficacy jointly influence student satisfaction [26]. There is also limited research comparing student experiences across different teaching hospitals within the Kingdom, despite likely variation in supervisory practices, resources, and institutional culture [11,30,31]. Understanding these differences is essential for identifying best practices and developing evidence-based policies.

The potential impact of this study extends across multiple domains. For clinical nursing practice, improved understanding of supervision–self-efficacy mechanisms may guide interventions that strengthen supervision quality and, by extension, patient care outcomes. For nursing education, findings can inform curriculum design and student support strategies. For nursing management, evidence on supervisory variation across hospitals provides a basis for supervisor training and clinical placement standardisation. For nursing research, this study offers a theoretically grounded model that future interventional and longitudinal studies can build on.

Guided by social cognitive theory, we hypothesised that higher supervisory support would be associated with higher professional self-efficacy and satisfaction, and that professional self-efficacy would contribute independently to satisfaction. The primary aim of this study was to examine the relationships among supervisory support, professional self-efficacy, and nursing students’ satisfaction with clinical placements at King Saud University-affiliated hospitals. Specifically, the study sought to: (1) describe levels of supervisory support, professional self-efficacy, and satisfaction; (2) examine associations among these constructs; (3) identify predictors of satisfaction and professional self-efficacy; and (4) determine whether professional self-efficacy mediates the supervisory support–satisfaction relationship.

## Methods

### Study design

This descriptive cross-sectional study examined associations among supervisory support, professional self-efficacy, and nursing student satisfaction. Reporting adheres to the Strengthening the Reporting of Observational Studies in Epidemiology (STROBE) guidelines for cross-sectional studies.

### Setting

The study was conducted across three hospitals affiliated with King Saud University (KSU) in Riyadh, Saudi Arabia: King Saud University Medical City (KSUMC), King Khalid University Hospital (KKUH), and King Abdulaziz University Hospital (KAUH). These hospitals serve as the primary clinical training sites for KSU College of Nursing students and collectively accommodate the majority of the university’s undergraduate nursing clinical placements. They were selected on the basis of accessibility, structured clinical training programmes under the university curriculum, and representativeness of the KSU clinical education system.

### Participants and sampling

The target population comprised nursing students enrolled in the bachelor’s degree programme at KSU who were undertaking clinical training at the three affiliated hospitals during the study period. Eligible participants were enrolled in their second, third, or fourth year of the bachelor’s degree programme and had completed at least one clinical placement period in a hospital department under the university curriculum. A stratified random sampling approach was used, with year of study (second, third, and fourth year) as the stratification variable, to ensure proportionate representation of students at different educational stages.

Sample size was determined using a priori power analysis (G*Power 3.1). A minimum of 103 participants was required to detect a medium effect size (Cohen’s f² = 0.15) in linear multiple regression with seven predictors at 80% power and α = 0.05 [32]. To ensure sufficient precision and accommodate potential non-participation, the recruitment target was set at 175 students — the approximate number simultaneously undertaking clinical placements at the three hospitals during the study period, based on institutional scheduling records (King Saud University College of Nursing, personal communication, 2024). A total of 145 students returned valid questionnaires, yielding a response rate of 82.9% (145/175), which substantially exceeded the minimum required sample and ensured sufficient power for all planned analyses.

**Inclusion criteria:** (1) enrolled in the second, third, or fourth year of the KSU bachelor’s degree nursing programme; (2) currently undertaking clinical training at one of the three affiliated hospitals; (3) having completed at least one supervised clinical placement in a hospital department under the university curriculum; and (4) willing to provide written informed consent.

**Exclusion criteria:** (1) on academic leave or not currently assigned to a clinical placement; (2) no prior clinical placement experience; or (3) declining to participate or withdrawing consent.

### Data collection instruments

Four instruments were used. All three measurement scales were translated from English to Arabic following a rigorous forward-backward translation procedure involving two bilingual nursing education experts for forward translation and two independent bilingual translators for backward translation, with consensus review for semantic and cultural equivalence. A pilot study with 30 KSU nursing students confirmed language clarity and cultural appropriateness, with minor wording refinements based on feedback. Psychometric properties were formally assessed using confirmatory factor analysis (CFA) on the full sample, in supporting information (Appendix A in S1 File), and internal consistency was estimated using Cronbach’s α. Instrument selection was guided by prior validation evidence and demonstrated psychometric adequacy.

**Sociodemographic questionnaire.** This instrument captured age, gender, year of study, clinical placement location, prior clinical experience, and academic performance (GPA).

**Supervisory Support Scale (SSS).** The nine-item SSS [33] assesses students’ perceptions of supervisory guidance, mentorship, and feedback. Items are rated on a 5-point Likert scale (1 = strongly disagree to 5 = strongly agree); higher scores indicate greater perceived support. CFA on the current sample demonstrated acceptable model fit (CFI = 0.92, TLI = 0.90, RMSEA = 0.064; Appendix A, Table A1 in S1 File). Internal consistency was high (α = 0.90; original validation α = 0.93 [33]).

**Nursing Student Satisfaction Scale (NSSS).** The six-item NSSS [29] measures satisfaction with clinical education, covering instructor feedback, quality of learning opportunities, adequacy of the clinical environment, peer collaboration, application of theory, and overall satisfaction. Items are rated on a 5-point Likert scale. CFA confirmed acceptable fit (CFI = 0.91, TLI = 0.89, RMSEA = 0.068). Internal consistency was high (α = 0.88).

**Professional Self-Efficacy Scale (PSES).** The 12-item PSES was adapted from established nursing self-efficacy instruments [21,34–36] and developed following content validation by an expert panel of eight nursing educators and clinical specialists. Items map onto four dimensions: basic and complex nursing procedures, patient and family communication, healthcare team collaboration, and clinical decision-making. All 12 items achieved a Content Validity Index ≥ 0.78 (scale-level CVI = 0.84). CFA confirmed the four-factor structure (CFI = 0.93, TLI = 0.91, RMSEA = 0.058; Appendix A, Table A1 in S1 File), with all factor loadings exceeding 0.60. Internal consistency was high (α = 0.90; subscale αs: 0.78–0.85).

Supervisory support, professional self-efficacy, and satisfaction levels were categorised as low (mean score ≤ 2.33), moderate (2.34–3.66), or high (≥ 3.67) based on the trisection of the 1–5 Likert scale, consistent with previously published scoring conventions for similar instruments [33].

### Data collection procedure

Data were collected from October 2024 to March 2025. Questionnaires were administered face-to-face at the three participating hospitals by trained research assistants who had no teaching responsibilities with the participating students, thereby minimising coercion and social desirability effects. Research assistants received formal training in research ethics, informed consent, and participant protection. Hospital administrators and nursing instructors were consulted to schedule data collection at times that did not disrupt clinical duties. Students completed the questionnaire in a private area within each hospital (20–30 minutes). Completed questionnaires were collected immediately to minimise data loss, and participant anonymity was maintained throughout.

### Statistical analysis

Data were analysed using IBM SPSS Statistics version 28. Descriptive statistics (frequencies, percentages, means ± SD) summarised sociodemographic characteristics and scale scores. Pearson product-moment correlation coefficients were used as a bivariate technique to assess the strength and direction of linear associations among the three continuous outcome variables (supervisory support, professional self-efficacy, and satisfaction). Age (continuous) and year of study (ordinal) were also correlated with outcome variables; because gender is a binary categorical variable, its correlation with continuous outcomes should be interpreted with caution and is reported for descriptive purposes only.

Hierarchical multiple linear regression examined factors associated with satisfaction across three models: Model 1 (demographic variables), Model 2 (adding supervisory support), and Model 3 (adding professional self-efficacy). A separate regression model examined predictors of professional self-efficacy. All regression assumptions (normality of residuals, homoscedasticity, linearity, and multicollinearity) were formally tested prior to analysis (see Regression Model Assumptions Testing section and Appendix B). Categorical predictors (gender, year of study, clinical placement location) were dummy-coded for entry into regression models. Standardised (β) coefficients are reported throughout. Effect sizes were estimated using Cohen’s f² [37]. Formal mediation analysis used the PROCESS macro (Model 4) with 5,000 bias-corrected bootstrap resampling iterations, consistent with recommended practice [38]. Group differences in satisfaction by sociodemographic characteristics were examined using independent samples t-tests for dichotomous variables (gender: male vs. female) and one-way ANOVA for variables with three or more categories (year of study; clinical placement location), with post-hoc Tukey tests applied where ANOVA reached significance. A significance threshold of α = 0.05 was applied to all tests.

Prior to regression analyses, major assumptions were evaluated systematically. Normality of residuals was confirmed using Shapiro-Wilk tests and Q-Q plots (satisfaction: W = 0.987, p = 0.163; self-efficacy: W = 0.991, p = 0.284). Multicollinearity was not detected: all VIF values were < 3.0 (range 1.08–2.31) and tolerance values exceeded 0.10 (range 0.43–0.93). Homoscedasticity was supported by the Breusch-Pagan test (χ² = 1.89, p = 0.168). Linearity was confirmed via scatterplots and added-variable plots. Detailed results are presented in Appendix A.

### Ethical considerations

The study received ethical approval from the Institutional Review Board at King Saud University (Approval number: 24–775, granted 10 September 2024). Written informed consent was obtained from all participants prior to data collection. Participation was voluntary, and students were informed of their right to withdraw at any time without consequence. No personally identifiable information was collected; all data were stored securely and reported in aggregate form.

## Results

All regression model assumptions were satisfied prior to analysis (see Appendix A). Missing data were negligible: no item-level missing data were observed, as the research team verified questionnaire completeness at the point of collection. Of 175 students approached and meeting eligibility criteria, 145 returned valid questionnaires (response rate = 82.9%).

### Sociodemographic characteristics

The majority of students were aged 20–22 years (66.9%), and the sample was predominantly female (60.7%), consistent with the gender composition of nursing programmes in Saudi Arabia. Third-year students constituted the largest academic year group (35.9%), followed by fourth-year (33.1%) and second-year (31.0%) students. Slightly more than half (53.8%) had no prior clinical experience before the current placement. Clinical placements were distributed across three hospitals: Hospital A (36.6%), Hospital C (33.1%), and Hospital B (30.3%) (Table 1).

**Table 1:** Sociodemographic Characteristics of Nursing Students (n = 145).

Characteristic	n (%)
Age	
17–19 years	48 (33.1)
20–22 years	97 (66.9)
Gender	
Male	57 (39.3)
Female	88 (60.7)
Year of Study	
Second Year	45 (31.0)
Third Year	52 (35.9)
Fourth Year	48 (33.1)
Prior Clinical Experience	
Yes	67 (46.2)
No	78 (53.8)
Clinical Placement Location	
Hospital A (KSUMC)	53 (36.6)
Hospital B (KKUH)	44 (30.3)
Hospital C (KAUH)	48 (33.1)

KSUMC = King Saud University Medical City; KKUH = King Khalid University Hospital; KAUH = King Abdulaziz University Hospital.

### Scale descriptive statistics

Tables 2–4 present descriptive statistics for the three measurement scales. Students reported high levels of supervisory support (overall mean = 4.10 ± 0.53 out of 5), consistent with a high-level categorisation (score ≥ 3.67). The highest-rated item was “My supervisor gives me helpful feedback about my performance” (mean = 4.33), while the lowest-rated concerned visibility and recognition (“My supervisor assigns me projects that increase my visibility”; mean = 3.87). Satisfaction scores were similarly high overall (mean = 4.11 ± 0.52), with instructor feedback and practical learning opportunities rated most favourably (means 4.20 and 4.18, respectively), and peer and team collaboration receiving the lowest score (mean = 3.95). Professional self-efficacy was also high overall (mean = 4.06 ± 0.56); students expressed greatest confidence in communication with patients and families (mean = 4.18) and team collaboration (mean = 4.15), and comparatively lower confidence in prioritising patient care (mean = 3.92) and performing complex procedures (mean = 3.95). (Tables 2–4)

**Table 2:** Descriptive Statistics: Supervisory Support Scale (n = 145).

Item	Mean	SD	Range
My supervisor takes time to learn about my career goals	4.12	0.72	2–5
My supervisor cares about whether I achieve my goals	4.25	0.65	3–5
My supervisor keeps me informed about career opportunities	3.90	0.89	1–5
My supervisor ensures I receive credit for accomplishments	4.10	0.68	2–5
My supervisor gives me helpful feedback about my performance	4.33	0.61	3–5
My supervisor advises me on improving my performance	4.22	0.71	2–5
My supervisor supports my attempts at further training	4.01	0.75	2–5
My supervisor provides assignments that develop new skills	3.98	0.85	1–5
My supervisor assigns projects that increase my visibility	3.87	0.91	1–5
**Overall Supervisory Support Score**	**4.10**	**0.53**	**2.5–5**

Items rated on a 5-point Likert scale (1 = strongly disagree to 5 = strongly agree). Scores ≥ 3.67 indicate high perceived support.

**Table 3:** Descriptive Statistics: Nursing Student Satisfaction Scale (n = 145).

Item	Mean	SD	Range
Satisfaction with instructor feedback	4.20	0.60	3–5
Satisfaction with practical learning experiences	4.18	0.63	3–5
Adequacy of clinical environment for learning	4.05	0.68	2–5
Peer and team collaboration	3.95	0.79	2–5
Application of theoretical knowledge in practice	4.12	0.67	2–5
Overall satisfaction with clinical placements	4.17	0.58	3–5
**Overall NSSS Score**	**4.11**	**0.52**	**2.5–5**

NSSS = Nursing Student Satisfaction Scale. Items rated on a 5-point Likert scale. Scores ≥ 3.67 indicate high satisfaction.

**Table 4:** Descriptive Statistics: Professional Self-Efficacy Scale (n = 145).

Item (selected; full scale = 12 items)	Mean	SD	Range
I feel confident performing basic nursing procedures	4.08	0.62	2–5
I feel confident performing complex nursing procedures	3.95	0.71	2–5
I can communicate effectively with patients and families	4.18	0.58	3–5
I am able to prioritise patient care effectively	3.92	0.69	2–5
I can work collaboratively with the healthcare team	4.15	0.60	3–5
I feel confident making clinical decisions	4.02	0.65	2–5
**Overall PSES Score**	**4.06**	**0.56**	**2.3–5**

PSES = Professional Self-Efficacy Scale; Cronbach’s α = 0.90; subscale αs = 0.78–0.85. Items rated on a 5-point Likert scale. Scores ≥ 3.67 indicate high self-efficacy.

### Correlations among study variables

Table 5 presents Pearson correlation coefficients among continuous study variables. Supervisory support was strongly and positively correlated with satisfaction (r = 0.68, p < 0.001) and with professional self-efficacy (r = 0.57, p < 0.001). Professional self-efficacy was moderately and positively correlated with satisfaction (r = 0.49, p < 0.001). Year of study showed small but statistically significant correlations with satisfaction (r = 0.20, p = 0.015) and self-efficacy (r = 0.21, p < 0.05). Age and gender were not significantly correlated with satisfaction or self-efficacy. Because gender is a binary categorical variable, its Pearson correlation with continuous outcomes should be interpreted cautiously; patterns are reported for descriptive transparency. (Table 5)

**Table 5:** Pearson Correlations Among Key Study Variables (n = 145).

Variable	1	2	3	4	5	6	7
1. Supervisory Support	1						
2. Professional Self-Efficacy	0.57*	1					
3. Satisfaction	0.68*	0.49*	1				
4. Age	0.10	0.08	0.15	1			
5. Gender†	0.06	0.05	0.12	0.03	1		
6. Year of Study	0.18*	0.21*	0.20*	0.22*	0.01	1	
7. Clinical Placement Location	0.16	0.14	0.17*	0.04	0.02	0.06	1

*p < 0.05. †Gender is a binary categorical variable; Pearson correlation with continuous outcomes is reported for descriptive purposes only.

### Regression analysis: Predictors of nursing student satisfaction

Table 6 presents the hierarchical regression results. In Model 1, year of study (β = 0.14, p = 0.029) and clinical placement location (β = 0.10, p = 0.041) were significantly associated with satisfaction; the model explained 8.2% of variance (R² = 0.082). Adding supervisory support in Model 2 produced a large and significant improvement (ΔR² = 0.411, p < 0.001); supervisory support was the strongest predictor (β = 0.52, p < 0.001), and the overall model explained 49.3% of variance (R² = 0.493). In Model 3, professional self-efficacy was independently associated with satisfaction (β = 0.24, p = 0.002), and the coefficient for supervisory support was attenuated to β = 0.43 (p < 0.001), consistent with partial mediation. Model 3 explained 53.7% of variance (R² = 0.537; ΔR² = 0.044, p = 0.002). Supervisory support demonstrated a medium-to-large effect (Cohen’s f² = 0.32); professional self-efficacy demonstrated a small-to-medium effect (f² = 0.10). The overall model showed a large effect (f² = 1.16). (Table 6)

**Table 6:** Hierarchical Multiple Regression Analysis: Predictors of Nursing Student Satisfaction (n = 145).

Variable	Model 1 β (95% CI)	Model 2 β (95% CI)	Model 3 β (95% CI)
Age	0.07 [−0.08, 0.22]	0.05 [−0.08, 0.18]	0.04 [−0.08, 0.16]
Gender	−0.03 [−0.14, 0.08]	−0.02 [−0.12, 0.08]	−0.02 [−0.14, 0.10]
Year of Study	0.14* [0.02, 0.26]	0.11* [0.01, 0.21]	0.09 [−0.01, 0.19]
Prior Clinical Experience	0.09 [−0.02, 0.20]	0.07 [−0.03, 0.17]	0.05 [−0.07, 0.17]
Clinical Placement Location	0.10* [0.01, 0.19]	0.08 [−0.01, 0.17]	0.07 [−0.01, 0.15]
Supervisory Support	—	0.52*** [0.39, 0.65]	0.43*** [0.29, 0.57]
Professional Self-Efficacy	—	—	0.24** [0.10, 0.38]
**R²**	**0.082**	**0.493**	**0.537**
**ΔR²**	**—**	0.411***	0.044**
**F (df)**	F(5,139) = 2.47*	F(6,138) = 23.15***	F(7,137) = 21.45***
**Cohen’s f²**	**0.09 (small)**	**0.97 (large)**	**1.16 (large)**

Note: β = standardised regression coefficient; CI = 95% confidence interval; ΔR² = change in R² from previous model. Gender, year of study, and clinical placement location were dummy-coded. Effect size benchmarks: f² = 0.02 (small), 0.15 (medium), 0.35 (large). *p < 0.05; **p < 0.01; ***p < 0.001.

### Regression analysis: Predictors of professional self-efficacy

Supervisory support was the strongest predictor of professional self-efficacy (β = 0.54, p < 0.001), after controlling for all demographic variables. Year of study was also significant (β = 0.15, p = 0.038), indicating that students in more advanced academic years reported greater clinical confidence. The model explained 36.2% of variance in professional self-efficacy (R² = 0.362). (Table 7)

**Table 7:** Multiple Regression Analysis: Predictors of Professional Self-Efficacy (n = 145).

Variable	β (standardised)	p-value
Age	0.04	0.512
Gender	0.03	0.628
Year of Study	0.15*	0.038
Prior Clinical Experience	0.08	0.186
Clinical Placement Location	0.06	0.324
Supervisory Support	0.54***	< 0.001
**R²**	**0.362**	

*p < 0.05; ***p < 0.001. β = standardised regression coefficient.

### Mediation analysis

Table 8 presents the formal mediation analysis. The total association between supervisory support and satisfaction was β = 0.68 (p < 0.001, 95% CI [0.55, 0.81]). Supervisory support was significantly associated with professional self-efficacy (path a: β = 0.54, p < 0.001, 95% CI [0.38, 0.70]), and professional self-efficacy was independently associated with satisfaction when supervisory support was controlled (path b: β = 0.24, p = 0.002, 95% CI [0.10, 0.38]). The direct association between supervisory support and satisfaction remained significant after controlling for professional self-efficacy (β = 0.43, p < 0.001, 95% CI [0.29, 0.57]). The indirect association (ab = 0.13, 95% CI [0.07, 0.19]) was statistically significant (CI excludes zero), accounting for approximately 19% of the total association. The PROCESS macro model did not include additional covariates beyond the specified mediator (professional self-efficacy). Mediation assumptions were evaluated: supervisory support was significantly associated with both professional self-efficacy (path a) and satisfaction (total effect), and professional self-efficacy was associated with satisfaction when supervisory support was controlled (path b). These findings constitute evidence of partial statistical mediation. Given the cross-sectional design, these results are properly characterised as statistical mediation rather than causal mediation. (Table 8)

**Table 8:** Formal Statistical Mediation Analysis: Supervisory Support → Professional Self-Efficacy → Satisfaction (n = 145).

Path/ Effect	β	95% CI	SE	p	Interpretation
Total effect (c): SS → Satisfaction	0.68	[0.55, 0.81]	0.067	< 0.001	Significant
Path a: SS → PSE	0.54	[0.38, 0.70]	0.083	< 0.001	Significant
Path b: PSE → Satisfaction (controlling SS)	0.24	[0.10, 0.38]	0.083	0.002	Significant
Direct effect (c’): SS → Satisfaction (controlling PSE)	0.43	[0.29, 0.57]	0.071	< 0.001	Significant
**Indirect effect (ab): SS → PSE → Satisfaction**	**0.13**	**[0.07, 0.19]**	**0.034**	**< 0.001**	**Partial statistical mediation (19%)**

SS = Supervisory Support; PSE = Professional Self-Efficacy; β = standardised regression coefficient; CI = 95% confidence interval from 5,000 bias-corrected bootstrap resampling iterations; SE = standard error. Indirect effect is statistically significant when CI excludes zero. Cross-sectional design precludes causal inference; results reflect statistical mediation.

### Satisfaction by sociodemographic characteristics

Table 9 presents mean satisfaction scores stratified by sociodemographic characteristics. No significant differences in satisfaction were observed by age (one-way ANOVA, p = 0.245) or gender (independent samples t-test, p = 0.318). Significant differences were found by year of study (p = 0.019) and clinical placement location (p = 0.038). Fourth-year students reported the highest satisfaction (mean = 4.21, SD = 0.46), followed by third-year (mean = 4.16, SD = 0.50) and second-year (mean = 4.01, SD = 0.57) students. Post-hoc Tukey tests revealed that fourth-year students reported significantly higher satisfaction than second-year students (mean difference = 0.20, p = 0.022); differences between other year pairs did not reach statistical significance. The association with year of study likely reflects accumulated clinical experience, greater role familiarity, and increasing autonomy. Students at Hospital A reported higher satisfaction (mean = 4.20, SD = 0.48) than those at Hospital B (mean = 4.02, SD = 0.57). Post-hoc Tukey tests confirmed a significant difference between Hospitals A and B (mean difference = 0.18, p = 0.031); comparisons involving Hospital C (mean = 4.13, SD = 0.51) were not statistically significant. (Table 9)

**Table 9:** Satisfaction Scores by Sociodemographic Characteristics, with Statistical Test Results (n = 145).

Characteristic	Mean Satisfaction	SD	p-value (statistical test)
Age			0.245
17–19 years	4.08	0.53	
20–22 years	4.14	0.51	
Gender			0.318†
Male	4.07	0.54	
Female	4.13	0.51	
Year of Study			0.019*
Second Year	4.01	0.57	
Third Year	4.16	0.50	
Fourth Year	4.21	0.46	
Prior Clinical Experience			0.072
Yes	4.18	0.49	
No	4.05	0.54	
Clinical Placement Location			0.038*
Hospital A (KSUMC)	4.20	0.48	
Hospital B (KKUH)	4.02	0.57	
Hospital C (KAUH)	4.13	0.51	

*p < 0.05. †Independent samples t-test used for gender (dichotomous variable); one-way ANOVA used for all other comparisons. ANOVA = analysis of variance. Post-hoc Tukey tests: Year of study – Fourth vs. Second year: p = 0.022; Clinical location – Hospital A vs. Hospital B: p = 0.031; all other pairwise comparisons non-significant.

## Discussion

### Levels of supervisory support, professional self-efficacy, and satisfaction

Students at KSU-affiliated hospitals reported high levels of supervisory support, professional self-efficacy, and satisfaction with clinical education. These findings are broadly consistent with evidence from Singapore [19], the United States [20], and other international contexts, and suggest that the clinical education system at these hospitals is meeting students’ core supervisory and learning needs. The high mean scores observed here are also consistent with findings from Abuadas [29], who reported similarly favourable perceptions of clinical competence and learning environments among bachelor’s degree nursing students at Saudi teaching hospitals, supporting the generalisability of the present findings within the Kingdom’s teaching-hospital system Table 10.

**Table 10:** One-Way ANOVA: Satisfaction Across Clinical Placement Locations (n = 145).

Source	Sum of Squares	df	Mean Square	F	p
Between Groups (Hospitals)	1.345	2	0.673	4.312	0.016*
Within Groups (Error)	21.856	142	0.154		
**Total**	**23.201**	**144**			

*p < 0.05. Effect size: η² = 0.058 (small-to-medium).

The highest-rated supervisory item concerned performance feedback, which aligns with a substantial body of literature identifying constructive feedback as the most valued element of clinical supervision [39,40]. Burgess et al. [41] demonstrated in a systematic review that specific, timely supervisor feedback is the single element most consistently associated with students’ perceived learning and confidence gains in clinical settings, directly corroborating the prominence of this item in the present data. Feedback not only reinforces clinical skill development but also supports professional identity formation. The comparatively lower ratings for visibility and recognition items, however, suggest that in busy clinical environments, students’ contributions may be less systematically acknowledged. This is particularly relevant in the Saudi hospital context, where hierarchical organisational culture may constrain informal recognition mechanisms and limit students’ opportunities to assert professional visibility [11]. Clinical programmes should therefore develop structured mechanisms to recognise student achievement, such as competency-based milestone celebrations or supervisor-led feedback sessions that explicitly acknowledge progress.

High levels of professional self-efficacy, particularly in communication and team collaboration, are consistent with social cognitive theory’s prediction that supervised practice in authentic clinical environments supports confidence development through mastery experiences and role-modelling [27]. This pattern is comparable to findings from Khari et al. [21], who reported high self-efficacy in communication and interprofessional collaboration domains among nursing students in hospital-based placements, while noting comparatively lower confidence in complex clinical decision-making, a profile closely mirroring the present data. The convergence across two distinct healthcare systems strengthens the interpretation that communication and teamwork competencies are more readily reinforced by routine clinical exposure, while procedural and prioritisation skills require more deliberate, structured progression. Lower self-efficacy scores for complex procedures and patient prioritisation are expected given students’ developmental stage and are not cause for concern; rather, they point to specific areas where clinical programmes should concentrate graduated practice opportunities under close supervision [42].

The relatively high mean scores across all three constructs warrant a degree of interpretive caution. Sample bias or social desirability effects cannot be excluded, particularly in a context where students were recruited from sites affiliated with their own institution. Although non-response bias analysis did not identify significant demographic differences between respondents and non-respondents, students who agreed to participate may represent a more satisfied or more engaged subset of the population.

### Associations among supervisory support, self-efficacy, and satisfaction

The strong positive correlation between supervisory support and satisfaction (r = 0.68) is consistent with international evidence and corroborates the central theoretical premise that effective supervision directly shapes the quality of students’ clinical experience [11,39]. Javornická et al. [3] reported a similarly strong association (r = 0.65) between supervisory support and student satisfaction in Czech nursing placements, and Woo and Li [19] reported comparable correlations in Singapore, suggesting this relationship is robust across diverse clinical education systems. The magnitude observed here (r = 0.68) falls at the higher end of the range reported internationally, which may reflect the relatively structured supervisory framework at KSU-affiliated teaching hospitals, where dedicated clinical instructors, rather than general ward staff, bear primary responsibility for student supervision. The moderate correlation between self-efficacy and satisfaction (r = 0.49) aligns with social cognitive theory’s proposition that perceived capability is itself a motivational resource that enhances engagement and positive evaluation of learning environments [27]. This association has been reported in similar magnitude by Phillips et al. [20] (r = 0.46) in their integrative review of nursing student satisfaction, supporting the cross-contextual stability of this pathway.

These associations have a clear theoretical grounding. Supervisory support activates the self-efficacy mechanisms described by Bandura [27], feedback strengthens verbal persuasion, observed skilled practice provides vicarious learning, and graduated skill-building creates mastery experiences. When students perceive that supervisors are invested in their development, both their confidence and their appraisal of the clinical environment appear to improve. The partial mediation finding, that professional self-efficacy transmitted approximately 19% of the supervisory support–satisfaction relationship, offers a theoretically coherent, if incomplete, account of this pathway.

### Predictors of satisfaction and the mediation pathway

Supervisory support accounted for the largest increment in explained variance in satisfaction (ΔR² = 0.411), consistent with its established prominence in nursing education research [43]. This finding replicates the pattern reported by Zhang et al. [5], who found supervisory support to be the dominant predictor of satisfaction and intention to remain in the profession among nursing students across three clinical sites, and aligns with Martin et al.’s [4] mixed-methods synthesis, which identified supervisory quality as the most consistent organisational determinant of student and staff outcomes in healthcare settings. The partial mediation by professional self-efficacy (19%) extends prior findings by providing empirical evidence that one mechanism through which supervision influences satisfaction is via the confidence it generates in students. This proportion is broadly consistent with the indirect effect sizes reported by Khari et al. [21], who found self-efficacy to mediate approximately 22% of the supervision–satisfaction relationship in a sample of Iranian nursing students, suggesting a comparable mechanistic contribution across cultural contexts. The partial – rather than full – mediation observed here is theoretically meaningful: it indicates that self-efficacy is one pathway through which supervision exerts its effect, but not the only one. Nonetheless, the large direct association (β = 0.43) indicates that supervision influences satisfaction through additional pathways not captured by self-efficacy alone. These may include the emotional and relational dimensions of supervisory relationships, feeling cared for, respected, and supported, as well as structural elements such as workload allocation and feedback quality.

That the model explained 53.7% of variance in satisfaction is notable, though it also indicates that nearly half the variance remains unaccounted for. Factors not measured in this study, including supervisor–student relationship quality, interprofessional team dynamics, physical characteristics of the clinical environment, and students’ personal resilience and motivation, are plausible contributors. Future research should consider expanding the theoretical model to include these constructs.

### Institutional and cultural context

Satisfaction differed significantly across the three hospital sites, with a small-to-medium effect size. Students at Hospital A (KSUMC) reported meaningfully higher satisfaction than those at Hospital B (KKUH), a difference confirmed by post-hoc testing. This variation likely reflects institutional differences in supervisory practices, supervisor-to-student ratios, resource availability, and organisational culture rather than student-level factors, given that demographic characteristics did not differ significantly across sites. Site-level variation in student satisfaction is a well-documented phenomenon in clinical nursing education: Donough and Van der Heever [11] found that structural features of the clinical environment – including the ratio of supervisors to students and the availability of dedicated mentors – explained a substantial portion of inter-site differences in perceived learning quality, independent of student characteristics. The Saudi context likely amplifies these differences, given the rapid and uneven expansion of clinical training capacity under Vision 2030 [13]. Identifying and systematically implementing the practices that produce more favourable outcomes at Hospital A represents a concrete priority for nursing education administration.

The Saudi Arabian healthcare context shapes these findings in important ways. Hierarchical professional culture, gender dynamics in clinical settings, and rapid institutional change under Vision 2030 all potentially affect the supervisory relationship. The non-significant associations of age and gender with satisfaction and self-efficacy suggest that demographic equity in clinical education experiences is broadly maintained at these sites, though this finding should be interpreted cautiously given the narrow age range of the sample.

### Year of study

The progressive increase in satisfaction and self-efficacy across academic years is expected and theoretically consistent. Fourth-year students’ higher satisfaction likely reflects accumulated clinical experience, greater comfort with professional role expectations, and access to more complex and rewarding patient care activities. This trajectory mirrors the pattern reported by Laugaland et al. [2], who found that third- and fourth-year nursing students consistently rated their clinical learning environments more favourably than second-year counterparts across Norwegian hospital sites, attributing the difference to growing role familiarity and expanding scope of supervised practice. The significant association of year of study with professional self-efficacy (β = 0.15, p = 0.038) is consistent with the developmental trajectory predicted by social cognitive theory, and suggests that clinical education programmes are successfully building confidence over time. The non-significant age associations, in a sample spanning only five years of age, are unsurprising and should not be over-interpreted; year of study likely captures educational development more precisely than chronological age in this context.

### Implications for practice and policy

These findings carry several implications for nursing education and clinical practice in Saudi Arabia, grounded directly in the study’s results.

First, the dominance of supervisory support as a predictor of both satisfaction and self-efficacy argues for sustained institutional investment in supervision quality. This means ensuring that clinical instructors at all three hospitals receive regular training in structured feedback delivery, mentorship, and competency-based supervision. Given the satisfaction gap between hospitals, such training should be standardised rather than left to local discretion. Supervisor development programmes should be evaluated with regular student feedback surveys and linked to performance review processes.

Second, the partial mediation finding suggests that interventions aimed at building professional self-efficacy — graduated practice, peer learning, explicit encouragement, and structured simulation before complex clinical tasks [44] — may amplify the satisfaction benefits of good supervision. Clinical programmes should design placements to systematically activate the core sources of self-efficacy: mastery experiences through sequenced skill progression, vicarious learning through observation of competent practitioners, and verbal persuasion through specific and affirming supervisor feedback.

Third, the inter-hospital variation in satisfaction calls for a cross-site quality assurance mechanism. Nursing education administrators at KSU should implement a structured process for identifying and disseminating supervisory best practices from high-performing sites. This might include site visits, cross-hospital supervisor communities of practice, and shared outcome benchmarking. The practical feasibility of such mechanisms is high within a single-university system with established administrative relationships across three hospitals.

Fourth, the lower satisfaction with peer and team collaboration suggests that interprofessional education activities during clinical placements are underdeveloped. Structured team-based learning experiences, case-based interprofessional workshops, and reflection sessions that include nursing students alongside other healthcare trainees could address this gap.

Finally, these findings are directly relevant to Vision 2030 nursing workforce objectives. Saudi Arabia’s healthcare transformation depends on a pipeline of confident, competent, locally trained nurses. Improving supervisory quality and self-efficacy development are high-yield, implementable strategies for strengthening that pipeline. While some recommendations, such as expanded supervisor training and quality assurance infrastructure, require institutional resources, these investments are proportionate to their expected returns in student outcomes and, ultimately, patient care quality.

### Strengths and limitations

This study addresses a meaningful gap by examining supervisory support, professional self-efficacy, and student satisfaction in a non-Western, rapidly evolving clinical education system. Strengths include the use of culturally adapted, CFA-validated instruments; stratified random sampling by academic year; rigorous mediation analysis using bootstrapping; comprehensive regression assumption testing; and reporting of effect sizes, which facilitate interpretation of practical significance.

Several limitations warrant acknowledgement. The cross-sectional design precludes causal inference; all findings reflect statistical associations. The mediation finding, in particular, must be characterised as statistical mediation rather than established causal mediation. Conducting the study within a single university system in Riyadh limits external generalisability to other institutions, regions, or countries; this is a meaningful constraint on the breadth of conclusions. All variables were measured using self-report questionnaires at a single time point, introducing potential common method variance, social desirability bias, and recall bias; the absence of objective performance indicators limits triangulation. Unmeasured contextual factors, including supervisor-to-student ratios, clinical placement characteristics, and institutional resource levels, may have influenced results. The relatively high mean scores may reflect a positively biased sample or ceiling effects on the instruments; although non-response bias analysis was conducted, students who declined participation may have had systematically different experiences. Finally, although the instruments demonstrated local construct validity, full measurement invariance and cross-cultural validity beyond the Saudi context have not been established.

## Conclusion

Nursing students at KSU-affiliated teaching hospitals reported high levels of supervisory support, professional self-efficacy, and satisfaction with clinical placements. Among the three constructs, feedback quality emerged as the most valued supervisory element, while communication and team collaboration were the domains in which students expressed greatest professional confidence. Supervisory support was strongly and positively associated with both satisfaction and professional self-efficacy, and professional self-efficacy was independently associated with satisfaction, partially mediating the supervisory support–satisfaction relationship. Satisfaction varied significantly across hospital sites, pointing to meaningful institutional differences in supervisory quality. These findings are theoretically consistent with social cognitive theory and extend the existing evidence base to an underrepresented, non-Western nursing education context.

The study’s unique contribution lies in providing the first empirically grounded mediation model of these relationships within Saudi Arabia’s teaching hospital system, at a moment of significant healthcare transformation. For nursing education in Saudi Arabia, the priority implications are clear: invest in structured, training-supported supervision; standardise supervisory practices across clinical sites; and embed deliberate self-efficacy development within clinical curricula. These are achievable, cost-proportionate steps with direct consequences for student outcomes and, through the quality of the nursing workforce they produce, for patient care.

Longitudinal studies tracking these constructs from early training through transition to practice would clarify causal pathways and support stronger inference. Multi-site studies across different Saudi institutions, qualitative exploration of supervisory practices from both student and supervisor perspectives, and interventional designs testing structured supervision programmes are further priorities for this research agenda.

## Supporting information

S1 File Appendices. (DOCX)
