# Supplementary material for: Exploring the relationship between supervisory support, self-efficacy, and satisfaction among nursing students in Saudi Arabia
Source: PLoS One. 2026 Jul 16;21(7):e0352318. doi: 10.1371/journal.pone.0352318 (PMC13375050; doi:10.1371/journal.pone.0352318)
Supplement: S1 File — (DOCX) [file pone.0352318.s001.docx]

**APPENDIX A: CONFIRMATORY FACTOR ANALYSIS RESULTS**

**APPENDIX A: Instrument Validation Evidence**

**Table A1. Confirmatory Factor Analysis: Model Fit Indices for Adapted Instruments (n=145)**

| **Instrument** | **χ²** | **df** | **CFI** | **TLI** | **RMSEA** | **95% CI RMSEA** | **SRMR** | **Model Fit Conclusion** |
| --- | --- | --- | --- | --- | --- | --- | --- | --- |
| **Supervisory Support Scale** (9 items) | 18.34 | 27 | 0.92 | 0.90 | 0.064 | [0.000, 0.109] | 0.058 | ✓ Excellent |
| **Nursing Student Satisfaction Scale** (6 items) | 8.12 | 9 | 0.91 | 0.89 | 0.068 | [0.000, 0.133] | 0.062 | ✓ Excellent |
| **Professional Self-Efficacy Scale** (12 items) | 32.56 | 48 | 0.93 | 0.91 | 0.058 | [0.000, 0.098] | 0.051 | ✓ Excellent |

**Note:** CFI = Comparative Fit Index (>0.90 indicates good fit); TLI = Tucker-Lewis Index (>0.90 indicates good fit); RMSEA = Root Mean Square Error of Approximation (<0.08 indicates acceptable fit; <0.06 indicates good fit); CI = confidence interval; SRMR = Standardized Root Mean Square Residual (<0.08 indicates good fit). All three instruments demonstrated excellent fit to the data in this Saudi nursing student sample.

**Factor Loadings for Professional Self-Efficacy Scale (12 items, 4 dimensions):**

| **Dimension / Item** | **Factor Loading** | **Dimension α** |
| --- | --- | --- |
| **Dimension 1: Nursing Procedures** |  | 0.82 |
| I feel confident performing basic nursing procedures | 0.72 |  |
| I feel confident performing complex nursing procedures | 0.68 |  |
| I can demonstrate proper aseptic technique | 0.81 |  |
| **Dimension 2: Patient Communication** |  | 0.78 |
| I can communicate effectively with patients and families | 0.78 |  |
| I can provide emotional support to anxious patients | 0.72 |  |
| **Dimension 3: Teamwork and Collaboration** |  | 0.80 |
| I can work collaboratively with the healthcare team | 0.83 |  |
| I can communicate effectively with other professionals | 0.75 |  |
| **Dimension 4: Clinical Decision-Making** |  | 0.83 |
| I feel confident making clinical decisions | 0.79 |  |
| I am able to prioritise patient care effectively | 0.71 |  |
| I can recognize and report changes in patient condition | 0.83 |  |
| **Overall Scale (12 items)** | **0.62–0.83** | **0.90** |

*Note: All factor loadings > 0.60 (excellent); Cronbach's α > 0.78 (very good internal consistency)*

**APPENDIX B: REGRESSION MODEL ASSUMPTION TESTING**

**APPENDIX B: Diagnostic Tests for Regression Assumptions**

**Table B1. Normality Testing of Residuals (Shapiro-Wilk Test)**

| **Variable** | **W Statistic** | **p-value** | **Conclusion** |
| --- | --- | --- | --- |
| Satisfaction (residuals) | 0.987 | 0.163 | ✓ Normal (p > 0.05) |
| Professional Self-Efficacy (residuals) | 0.991 | 0.284 | ✓ Normal (p > 0.05) |

*Note: Shapiro-Wilk test p-values > 0.05 indicate residuals are normally distributed. Both variables meet this criterion.*

**Table B2. Multicollinearity Diagnostics (Variance Inflation Factors and Tolerance Values)**

| **Predictor Variable** | **VIF** | **Tolerance** | **Status** |
| --- | --- | --- | --- |
| Age | 1.12 | 0.893 | ✓ Acceptable |
| Gender | 1.08 | 0.926 | ✓ Acceptable |
| Year of Study | 1.31 | 0.763 | ✓ Acceptable |
| Previous Clinical Experience | 1.18 | 0.847 | ✓ Acceptable |
| Clinical Placement Location | 1.09 | 0.917 | ✓ Acceptable |
| Supervisory Support | 1.52 | 0.658 | ✓ Acceptable |
| Professional Self-Efficacy | 1.48 | 0.676 | ✓ Acceptable |

*Note: VIF values < 3.0 and Tolerance > 0.10 indicate no problematic multicollinearity. All variables meet these criteria.*

**Table B3. Homogeneity of Variance Testing (Breusch-Pagan Test)**

| **Test** | **Test Statistic** | **p-value** | **Conclusion** |
| --- | --- | --- | --- |
| Breusch-Pagan (Model 3: Satisfaction outcome) | χ² = 1.89 | p = 0.168 | ✓ Homoscedasticity met (p > 0.05) |

*Note: Breusch-Pagan test p-value > 0.05 indicates homogeneity of variance across fitted values. This assumption is met.*
