# Supplementary material for: Exploring the relationship between supervisory support, self-efficacy, and satisfaction among nursing students in Saudi Arabia
Source: PLoS One. 2026 Jul 16;21(7):e0352318. doi: 10.1371/journal.pone.0352318 (PMC13375050; doi:10.1371/journal.pone.0352318)
Supplement: Attachment — Submitted filename: Upload Reviewer Attachment.pdf [file pone.0352318.s004.pdf]

Overall, the manuscript presents a well-structured and comprehensive study addressing important constructs in nursing education, including supervisory support, professional self-efficacy, and student satisfaction. The use of validated instruments, appropriate statistical analyses, and a theoretically grounded framework are notable strengths. However, several methodological, analytical, and reporting issues require clarification and refinement to enhance the scientific rigor, consistency, and interpretability of the study. The following comments are provided to support the authors in improving the quality and clarity of the manuscript in line with international publication standards.

Abstract

- Please provide a more concise summary of the study background.
- The general objective of the study should be explicitly stated at the end of the background section to improve clarity and coherence of the abstract structure.
- Clearly state the study design in a complete form, e.g., "This descriptive cross-sectional study was conducted among 145 nursing students at King Saud University hospitals," ensuring consistency with standard reporting guidelines.
- The statement "Descriptive statistics summarized study variables" is too general. Specify the key study variables that were summarized (e.g., supervisory support, professional self-efficacy, and student satisfaction).
- The statement "Pearson correlations examined relationships" should be revised to reflect the purpose of correlation analysis more accurately, i.e., to assess the strength and direction of associations between quantitative variables.
- Key socio-demographic characteristics of the sample should be briefly presented before reporting the main study results to provide a better context for interpretation.
- Provide the response rate at the beginning of the Results section to enhance transparency regarding sample recruitment and data completeness.
- Clarify what the reported mean scores are based on (e.g., "out of 5") and ensure consistent reporting of means with standard deviations (±SD) throughout the abstract.
- Key regression results should be clearly and concisely stated. For example:
- "In regression analysis, supervisory support remained significantly associated with satisfaction ($\beta=0.43$, $p<0.001$) even after accounting for professional self-efficacy, consistent with partial statistical mediation".

Background

- Please include a dedicated paragraph discussing the key challenges and barriers related to supervisory support and professional self-efficacy in nursing education. This may include issues such as workload of clinical instructors, variability in supervision quality, limited feedback opportunities, and student–supervisor communication gaps, supported by relevant literature.
- The statement "Given its importance, the quality of supervisory support is paramount to ensuring the future success of nursing professionals [5]" should be further strengthened by providing concrete examples of how supervisory

support influences student outcomes (e.g., clinical skill development, confidence building, and transition to practice).

- Several paragraphs in the background section contain grammatical and stylistic issues. Please thoroughly revise the section to ensure consistency with formal academic writing standards, clarity of expression, and coherence across paragraphs.
- The statement “In this context, the role of clinical instructors who guide, mentor, and evaluate students in real-world healthcare settings becomes critically important [9]” should be further elaborated to highlight how these roles directly influence student learning outcomes, professional development, and clinical competence.
- The statement “Guided by social cognitive theory, we hypothesized that higher supervisory support would be associated with higher professional self-efficacy and satisfaction, and that professional self-efficacy would contribute independently to satisfaction” should be clearly positioned immediately before the general and specific objectives to improve logical flow and theoretical coherence.
- Clearly explain how Social Cognitive Theory guided the selection of study variables (supervisory support, professional self-efficacy, and satisfaction), particularly how constructs such as observational learning, feedback, and mastery experience were operationalized in the study context.
- A dedicated paragraph should be added discussing the potential impact of the study on:
- Clinical nursing practice (e.g., improving supervision quality and patient care outcomes)
- Nursing education (e.g., curriculum design and student support strategies)
- Nursing management (e.g., supervisor training and clinical placement organization)
- Nursing research (e.g., future intervention studies and theoretical model testing)

Methods:

- Summarize the study design section more concisely while maintaining all essential methodological details, ensuring clarity and alignment with standard reporting guidelines (e.g., STROBE).
- The study setting should be revised to clearly specify the number and names of the hospitals affiliated with King Saud University in Riyadh. In addition, provide a clear justification for selecting these specific hospitals (e.g., accessibility, clinical training structure, or representativeness).
- The rationale for conducting the study within a single university should be explicitly stated. This represents a limitation in terms of external validity and generalizability, which should also be acknowledged in the Limitations section.
- The statement regarding increasing the sample size to account for non-response should be revised for clarity. Specify the assumed attrition rate (10–15%) and avoid repetition of this explanation across the Methods section.

- Report the total target population size with an updated reference before sample size calculation. Additionally, clearly describe the formula or statistical method used for sample size estimation, including proper citation.
- The description of stratification should be revised. Clearly explain the stratified random sampling procedure used to recruit participants, including strata definition and allocation process, rather than providing a conceptual justification only.
- The statement "students who had completed at least one clinical rotation" should be revised for clarity. Ensure correct terminology consistent with nursing education structure (e.g., clinical placements/clinical training in hospital departments under university curriculum).
- The inclusion criteria should explicitly include academic level (e.g., first, second, third, fourth year) and educational level (e.g., bachelor's degree program). These are essential eligibility variables and must be clearly defined.
- Specify the exact nature of the data collection tools (e.g., structured self-administered questionnaire, survey, or standardized checklist) to improve methodological transparency.
- The statement regarding the selection of tools based on validity and reliability is too general. Therefore, provide a clearer explanation of how psychometric properties (e.g., Cronbach's alpha, prior validation studies) informed instrument selection.
- The sentence explaining the subgroup analysis context is not essential for the Methods section and should be removed to improve focus and conciseness.
- Provide a detailed description of the translation and adaptation process, including:
- Number and expertise of experts involved
- Specific modifications made to questionnaire items
- Additionally, clarify why construct validity was assessed after data collection, as this should ideally be conducted during pilot testing before full-scale data collection.
- Clearly explain how levels (high, moderate, low) of supervisory support, satisfaction, and self-efficacy were determined (e.g., cut-off points, mean score ranges, or validated scoring frameworks).
- The data collection procedures are described more than once. Merge and present them under a single clear subheading to improve structure and avoid redundancy.
- The statement regarding non-response bias requires clarification. Clearly explain how comparisons between respondents and non-respondents were performed and what specific institutional data were used.
- The response rate should be recalculated and clearly reported based on the number of completed questionnaires relative to the sampled participants, not the total population size.
- The sentence describing data collection should be revised to formal academic language, clearly indicating that data were collected through face-to-face administration at selected hospital sites.

- The statistical analysis section should be summarized into one coherent paragraph, clearly describing all statistical tests used, significance thresholds, and software employed.
- The statement regarding correlation analysis is not accurate. Revise to clearly state that Pearson correlation was used as a bivariate statistical technique to assess the strength and direction of linear relationships between continuous variables only, not demographic categorical variables.

Results

- Report whether the assumptions for Pearson correlation, regression analysis, and ANOVA (e.g., normality, linearity, homoscedasticity, multicollinearity) were assessed before conducting the analyses.
- Clarify the rationale for including demographic variables such as age and gender in the correlation analysis. Since these variables are categorical or limited in variability, the appropriateness of using correlation with these variables should be justified, and alternative statistical approaches may be more suitable.
- Write the name of the table at the end of each paragraph as an example (Table 1).
- Table numbering appears duplicated (e.g., Table 9 is repeated twice). Revise the numbering for consistency and clarity.
- Provide a concise narrative summary for each table. Each table should be accompanied by one or two well-developed paragraphs highlighting the most important findings and statistically significant results.
- Ensure that the interpretation of correlation coefficients follows accepted statistical interpretation guidelines.
- Some statements contain interpretation or explanation rather than pure reporting of findings (e.g., “likely due to increased familiarity…”). These interpretations should be moved to the Discussion section.
- Based on the cross-sectional design, the mediation findings should be interpreted as statistical mediation rather than causal mediation.
- Explain how categorical variables such as gender, year of study, and clinical placement location were coded and entered into the regression models.
- Since the ANOVA showed significant differences across clinical placement locations, provide the post-hoc comparisons (e.g., Tukey test) to identify which groups differed significantly.
- Some statistical findings reported in the narrative are not fully aligned with the tables. So, carefully verify consistency between text, tables, beta coefficients, p-values, and confidence intervals.
- Clarify whether standardized or unstandardized coefficients are reported.
- The regression tables report β coefficients, but it is unclear whether these are standardized or unstandardized estimates. Specify this clearly in the table notes.
- Several findings are repeated multiple times in both the text and tables, particularly regarding supervisory support and satisfaction. Try to reduce redundancy to improve readability and conciseness.

- The mediation analysis is well presented; however, explicitly state whether mediation assumptions were assessed and whether the PROCESS macro model included covariates in all pathways.
- Report effect sizes for all major statistical analyses (e.g., correlations, ANOVA, regression) to improve the interpretation of practical significance.
- Provide whether there were any missing data and describe how missing values were managed during analysis.
- Some tables are overly dense and may benefit from formatting improvements, including alignment of decimal places and simplification of repetitive information.
- Since the study appears to use a cross-sectional design, terms implying causality such as “influencing,” “effect,” or “predictors” should be used cautiously and replaced with “associated with” where appropriate.

Discussion

- The Discussion section should focus on interpretation and contextualization of findings rather than restating detailed statistical results and numerical values (e.g., means, correlation coefficients, p-values, and beta coefficients), as these have already been fully presented in the Results section. Instead, the emphasis should be placed on explaining the meaning, implications, and theoretical relevance of the findings.
- Since the study employed a cross-sectional design, causal language such as “influence,” “predictor,” or “fosters” should be interpreted cautiously and replaced with more appropriate associative terminology where necessary.
- Several findings are discussed descriptively without sufficient comparison to regional or international studies. Expand the comparison with previous research and clarify similarities or discrepancies with existing evidence.
- Although Social Cognitive Theory is mentioned, the discussion would benefit from a more critical explanation of how the findings specifically support or extend the theoretical framework.
- The Discussion section repeatedly restates statistical values and findings already presented in the Results section. Focus more on interpretation and implications rather than repeating numerical outcomes.
- The discussion should more clearly explain how cultural, educational, and institutional characteristics in Saudi Arabia may influence supervisory support, self-efficacy, and student satisfaction.
- The observed differences between hospitals are mentioned briefly. Provide a more in-depth discussion of possible institutional or educational factors contributing to these differences.
- The Discussion section should acknowledge potential biases such as self-report bias, social desirability bias, and unmeasured confounding variables that may have influenced the findings.

- Although mediation analysis was conducted, the findings should be described as evidence of “statistical mediation” rather than causal mediation due to the cross-sectional nature of the study.
- The implications for nursing educators, clinical supervisors, and policymakers should be expanded with more specific and actionable recommendations based on the findings.
- The Discussion section would benefit from a more comprehensive acknowledgment of study limitations, including the single-university setting, convenience sampling, cross-sectional design, and limited generalizability of the findings.
- Some statements appear overly conclusive given the observational design and self-reported measures. Please moderate claims to avoid overgeneralization.
- The discussion briefly mentions non-significant findings (e.g., age and gender) without critically interpreting why these variables may not have been associated with satisfaction or self-efficacy.
- Although the regression model explained 53.7% of the variance in satisfaction, a substantial proportion of variance remains unexplained. Discuss other potential factors that may contribute to nursing student satisfaction.
- Since professional self-efficacy mediated only 19% of the relationship, the discussion should further elaborate on other possible mediating mechanisms underlying the supervisory support–satisfaction relationship.
- Recommendations for improving supervisory support are valuable; however, the discussion should address the practical feasibility and resource implications of implementing these strategies in clinical settings.
- Some recommendations appear broad and insufficiently linked to the actual study findings. So, ensure that all recommendations are directly supported by the presented results.
- Several paragraphs discuss findings separately without sufficient synthesis across themes. Improve the overall flow and integration of the discussion.

Implications of the study

- Some recommendations appear broad and general. Ensure that all educational and practice implications are directly and explicitly linked to the study findings.
- Given the cross-sectional design, recommendations regarding supervision and self-efficacy development should be presented more cautiously and framed as potential strategies rather than confirmed solutions.
- The implications section would benefit from more specific examples of how nursing programs and healthcare institutions can implement supportive supervision and self-efficacy enhancement strategies in practice.
- Recommendations such as supervisor training and quality assurance standardization may require substantial institutional resources. Discuss the practical feasibility and potential challenges of implementation.
- The connection with Saudi Vision 2030 is relevant; however, this section would benefit from a more detailed explanation of how the study findings specifically contribute to national nursing workforce development and healthcare transformation goals.

Strengths and Limitations

- Although stratified random sampling was used, the study was still conducted within a single university system in Riyadh. Emphasize that this limits external generalizability to other regions or institutions in Saudi Arabia and internationally.
- Since all variables were measured using self-report questionnaires at a single time point, the risk of common method variance should be explicitly acknowledged and discussed as a key methodological limitation.
- While CFA and instrument validation are strengths, clarify whether full measurement invariance or cross-cultural validation was tested, especially given the adapted nature of the instruments.
- The relatively high mean scores may indicate possible response bias or sample bias. This should be explicitly discussed, including whether non-response bias was assessed and how it might have affected the results.

Conclusion

- The conclusion largely restates findings already presented in the Results and Discussion sections. Focus more on synthesis and overarching contribution rather than repeating statistical relationships.
- Some phrases (e.g., "supervisory support may enhance learning") should be carefully framed to avoid implying causality, given the cross-sectional design of the study.
- The conclusion would benefit from a clearer statement highlighting the unique contribution of the study to nursing education literature in the Saudi or regional context.
- The recommendations for practice and policy are valuable but slightly extensive for a conclusion. Condense and prioritize the most critical implications.
- While longitudinal studies are appropriately recommended, it would strengthen the conclusion to also suggest specific future directions (e.g., interventional studies, multi-site studies, or qualitative exploration of supervisory practices).
