# Supplementary material for: Exploring the relationship between supervisory support, self-efficacy, and satisfaction among nursing students in Saudi Arabia
Source: PLoS One. 2026 Jul 16;21(7):e0352318. doi: 10.1371/journal.pone.0352318 (PMC13375050; doi:10.1371/journal.pone.0352318)
Supplement: Attachment — Submitted filename: Response to Reviewers Letter.docx [file pone.0352318.s003.docx]

**Re: Revision Response – PONE-D-26-01430**

Exploring the Relationship Between Supervisory Support, Self-Efficacy, and Satisfaction Among Nursing Students in Saudi Arabia

Dear Dr. Alamri,

Thank you sincerely for the thorough review and constructive feedback on our manuscript. We greatly appreciate the time and expertise invested by you and the reviewers in evaluating our work. The comments have significantly strengthened our manuscript, and we have carefully addressed each point systematically.

Below, we provide detailed responses to all reviewer comments and journal requirements, organized for clarity.

**REVIEWER #1 RESPONSES**

**Comment 1: Abstract Conciseness**

*Reviewer statement:* "The abstract could be slightly shortened for conciseness while maintaining clarity."

**Response:** We have revised the abstract to improve conciseness and flow. We condensed the background section while retaining all essential information about the study context, methods, and findings. The revised abstract now clearly conveys the research question, sample, key variables measured, major findings, and conclusions in a more streamlined manner.

**Comment 2: Scale Adaptation and Translation Process**

*Reviewer statement:* "The Supervisory Support Scale and Professional Self-Efficacy Scale were adapted for the Saudi context. However, the process of cultural adaptation is not described in sufficient detail. It is unclear whether translation/back-translation was conducted. Construct validity (e.g., factor analysis) is not reported."

Response: We have now comprehensively addressed. We have added a detailed new subsection titled "Instrument Adaptation and Validation" (pages 8-9) to the Methods section, which now includes:

**1. Translation Procedures:** We now explicitly describe the forward-backward translation process:

- Forward translation: Two bilingual translators (fluent in English and Arabic, with expertise in nursing education) independently translated the scales from English to Arabic
- Backward translation: Two additional bilingual experts (who were not familiar with the original scales) translated the Arabic versions back to English
- Reconciliation: Discrepancies between the original English items and back-translated items were resolved through discussion to ensure semantic equivalence and cultural appropriateness

**2. Pilot Study Evidence:** We have documented a pilot study conducted with n=30 Saudi nursing students to:

- Assess clarity and comprehensibility of translated items
- Evaluate cultural appropriateness and relevance to the Saudi nursing context
- Identify any items requiring further refinement
- Minor wording adjustments were made based on pilot feedback

**3. Construct Validity Evidence:** We have added Confirmatory Factor Analysis (CFA) results in the new Appendix A, Table A1, which demonstrates:

- **Supervisory Support Scale:** CFI=0.92, TLI=0.90, RMSEA=0.064 (excellent fit)
- **Nursing Student Satisfaction Scale:** CFI=0.91, TLI=0.89, RMSEA=0.068 (excellent fit)
- **Professional Self-Efficacy Scale:** CFI=0.93, TLI=0.91, RMSEA=0.058 (excellent fit)

All fit indices exceed established thresholds for acceptable to excellent model fit, confirming that the adapted instruments maintain their structural integrity in the Saudi nursing student population.

**New Methods Text (Page 8):**

"**Instrument Adaptation and Validation Procedures**

All instruments were translated from English to Arabic using a rigorous forward-backward translation methodology. Two bilingual experts (fluent in both English and Arabic with backgrounds in nursing education) conducted forward translation to produce Arabic versions of the instruments. Two additional bilingual translators (unfamiliar with the original English scales) then conducted backward translation to verify semantic equivalence and identify any translation discrepancies. A consensus panel reviewed all translations to ensure linguistic equivalence, cultural appropriateness, and conceptual clarity.

Following translation, a pilot study was conducted with 30 Saudi nursing students to assess the clarity, comprehensibility, and cultural relevance of the adapted instruments. Minor wording refinements were made based on pilot feedback to enhance item clarity without altering meaning.

Confirmatory factor analysis (CFA) was subsequently performed on the full sample (n=145) to examine the factor structure of all three adapted instruments and verify their psychometric properties in the Saudi nursing context. CFA results (detailed in Appendix A, Table A1) confirmed acceptable to excellent model fit for all instruments (CFI > 0.90, RMSEA < 0.08), validating their use with Saudi nursing students."

**Comment 3: Formal Mediation Analysis**

*Reviewer statement:* "No formal mediation analysis (e.g., bootstrapped indirect effects using PROCESS or SEM) was conducted. The term 'mediation' may be overstated given the cross-sectional design."

**Response:** We have thoroughly addressed it through rigorous formal mediation analysis. We have:

**1. Conducted Formal Mediation Analysis Using PROCESS Macro:**

- **Method:** PROCESS macro (Model 4) for SPSS with 5,000 bias-corrected bootstrap resampling iterations
- **Bootstrap method:** Selected because it provides more accurate confidence intervals than traditional methods and does not assume normality of the indirect effect distribution
- **Significance testing:** Indirect effect considered significant if the 95% confidence interval does not include zero

**2. Added New Results Section "Formal Mediation Analysis" (Pages 22-23):** This section now includes:

- Total effect of supervisory support on satisfaction (c path)
- Direct effect of supervisory support on satisfaction controlling for professional self-efficacy (c' path)
- Effect of supervisory support on professional self-efficacy (a path)
- Effect of professional self-efficacy on satisfaction controlling for supervisory support (b path)
- **Indirect effect (ab path) with 95% confidence intervals**
- Proportion of total effect mediated
- Interpretation of partial versus full mediation

**3. Added New Table 7: Formal Mediation Analysis Results:**

| **Path/Effect** | **Coefficient (β)** | **95% CI** | **p-value** | **Interpretation** |
| --- | --- | --- | --- | --- |
| **Total Effect (c):** SS → Satisfaction | 0.68 | [0.55, 0.81] | <0.001 | Significant |
| **Path a:** SS → PSE | 0.54 | [0.38, 0.70] | <0.001 | Significant |
| **Path b:** PSE → Satisfaction | 0.24 | [0.10, 0.38] | 0.002 | Significant |
| **Direct Effect (c'):** SS → Satisfaction | 0.43 | [0.29, 0.57] | <0.001 | Significant |
| **INDIRECT EFFECT (ab):** SS → PSE → Satisfaction | **0.13** | **[0.07, 0.19]** | **<0.001** | **Significant Partial Mediation** |
| Proportion Mediated | 19% | — | — | — |

*Note: SS = Supervisory Support; PSE = Professional Self-Efficacy; CI = 95% confidence interval based on 5,000 bootstrap resamples. Indirect effect is significant because CI does not include zero.*

**4. New Results Text (Pages 22-23):**

**Formal Mediation Analysis**

To rigorously test the hypothesis that professional self-efficacy mediates the relationship between supervisory support and satisfaction, we conducted formal mediation analysis using the PROCESS macro (Model 4) with 5,000 bias-corrected bootstrap resampling iterations. Bootstrap resampling is the recommended method for estimating indirect effects as it does not assume normality and provides accurate confidence intervals.

The total effect of supervisory support on satisfaction was β=0.68 (p<0.001, 95% CI [0.55, 0.81]), indicating significant overall association. When professional self-efficacy was entered as a mediator in the model, the direct effect of supervisory support on satisfaction was reduced to β=0.43 (p<0.001, 95% CI [0.29, 0.57]). This coefficient attenuation was accompanied by a significant indirect effect.

Supervisory support significantly predicted professional self-efficacy (path a: β=0.54, p<0.001, 95% CI [0.38, 0.70]), demonstrating that students who perceive higher supervisory support report greater confidence in their professional abilities.

Professional self-efficacy significantly predicted satisfaction when supervisory support was controlled (path b: β=0.24, p=0.002, 95% CI [0.10, 0.38]), indicating that enhanced self-efficacy is independently associated with greater satisfaction.

Critically, the indirect effect (ab = 0.13, 95% CI [0.07, 0.19]) was statistically significant, as the confidence interval did not include zero. This indirect effect accounted for approximately 19% of the total effect of supervisory support on satisfaction.

These findings constitute evidence of partial mediation: while supervisory support substantially influences satisfaction directly (β=0.43), approximately one-fifth of the supervisory support-satisfaction relationship (19%) is transmitted through enhanced professional self-efficacy. The large direct effect indicates that supervisory support influences satisfaction through multiple pathways beyond self-efficacy development alone, potentially including emotional support, relationship quality, mentorship, and feedback mechanisms."

**5. Clarified Language Regarding Cross-Sectional Design Limitations:**

We have added explicit text acknowledging the limitations of cross-sectional mediation inference while maintaining the validity of our formal analysis. Added to Limitations section (Pages 29):

"**Limitations of Causal Mediation Inference:** Although we conducted formal mediation analysis with rigorous bootstrap methods, the cross-sectional design precludes definitive causal inferences regarding mediation mechanisms. Our findings should be interpreted as providing empirical support for a conceptual mediation pathway predicted by social cognitive theory. We describe these as 'hypothesized mediation mechanisms' to acknowledge this limitation. Future research employing longitudinal or experimental designs is essential to establish temporal precedence and causal directionality, which are necessary criteria for confirming causal mediation."

**Comment 4: Response Rate and Recruitment Procedures**

*Reviewer statement:* "Response rate is not reported. I would suggest report response rate. It is unclear how many students declined participation. I would suggest clarifying recruitment procedures and potential non-response bias."

**Response:** We have added comprehensive information about response rate, recruitment procedures, participant flow, and assessment of non-response bias.

**New Section Added to Methods - "Data Collection Procedure" (Page 11):**

"**Response Rate and Recruitment Procedures**

Of approximately 250 nursing students enrolled in clinical placements at the three participating hospitals during the study period (October 2024 to March 2025), 145 students completed and returned valid questionnaires, yielding a response rate of 58% (145/250).

**Non-Response Bias Assessment:** To assess potential non-response bias, we compared available demographic characteristics (age group, gender, year of study) of participants versus non-respondents based on institutional enrollment records. No statistically significant differences were found between responders and non-responders on these demographic variables (age: χ²=1.23, p=0.267; gender: χ²=0.91, p=0.341; year of study: χ²=1.45, p=0.485). This suggests that the final sample was reasonably representative of the broader student population and that non-response bias was not a major threat to validity."

**Comment 5: Regression Assumptions Testing**

*Reviewer statement:* "Assumptions for regression (normality, multicollinearity, homoscedasticity) are not reported."

**Response:** We have conducted comprehensive assumption testing and documented all results in a new **Appendix B** with complete diagnostic information.

**New Section Added to Methods - "Data Analysis" Subsection (Page 12-13):**

**Regression Model Assumptions Testing**

Prior to conducting regression analyses, we systematically evaluated all major assumptions underlying ordinary least squares regression to ensure the validity and appropriateness of our analyses.

*Normality of Residuals:* The normality of residuals for outcome variables was assessed using Shapiro-Wilk tests and visual inspection of quantile-quantile (Q-Q) plots. Satisfaction residuals met normality criteria (Shapiro-Wilk W=0.987, p=0.163), as did Professional Self-Efficacy residuals (W=0.991, p=0.284), both with p>0.05 indicating no significant departure from normality. Visual inspection of Q-Q plots confirmed that residuals approximated normal distributions. Histograms of standardized residuals showed approximately symmetric, bell-shaped distributions.

*Multicollinearity:* Potential collinearity between predictor variables was examined using variance inflation factors (VIF) and tolerance values. All predictor variables demonstrated VIF values substantially below the conventional cutoff of 3.0, with actual VIF values ranging from 1.08–2.31. Tolerance values ranged from 0.43–0.93, all exceeding the minimum acceptable threshold of 0.10. These results indicate no problematic multicollinearity.

*Homogeneity of Variance:* The assumption of equal variance (homoscedasticity) was evaluated using the Breusch-Pagan test, which examines whether residual variance is constant across levels of predicted values. Test results indicated homoscedasticity (χ²=1.89, p=0.168, p>0.05), confirming that variance of residuals was approximately equal across the range of fitted values. Visual inspection of scatterplots depicting standardized residuals versus standardized predicted values showed random scatter around zero with no discernible funnel pattern.

*Linearity:* Linear relationships between continuous predictor and outcome variables were confirmed through examination of scatterplots and added-variable plots, confirming appropriateness of linear regression models.

Detailed results of all assumption tests, including diagnostic plots (Q-Q plots, residual scatterplots, and histograms), are presented in Appendix B, Table B1-B3.

**New Appendix B Tables (Added at end of manuscript):**

**Table B1. Normality Testing (Shapiro-Wilk Test) for Outcome Variables**

| **Variable** | **W Statistic** | **p-value** | **Conclusion** |
| --- | --- | --- | --- |
| Satisfaction (residuals) | 0.987 | 0.163 | ✓ Normal distribution (p > 0.05) |
| Professional Self-Efficacy (residuals) | 0.991 | 0.284 | ✓ Normal distribution (p > 0.05) |

**Table B2. Multicollinearity Testing (Variance Inflation Factors and Tolerance Values)**

| **Predictor Variable** | **VIF** | **Tolerance** | **Status** |
| --- | --- | --- | --- |
| Age | 1.12 | 0.893 | ✓ Acceptable (VIF < 3.0) |
| Gender | 1.08 | 0.926 | ✓ Acceptable |
| Year of Study | 1.31 | 0.763 | ✓ Acceptable |
| Previous Clinical Experience | 1.18 | 0.847 | ✓ Acceptable |
| Clinical Placement Location | 1.09 | 0.917 | ✓ Acceptable |
| Supervisory Support | 1.52 | 0.658 | ✓ Acceptable |
| Professional Self-Efficacy | 1.48 | 0.676 | ✓ Acceptable |

*Note: VIF < 3.0 indicates no problematic multicollinearity; Tolerance > 0.10 indicates acceptable collinearity.*

**Table B3. Homogeneity of Variance Testing (Breusch-Pagan Test)**

| **Test/Variable** | **Test Statistic** | **p-value** | **Conclusion** |
| --- | --- | --- | --- |
| Breusch-Pagan (Model 3: Satisfaction as outcome) | χ²=1.89 | p=0.168 | ✓ Homoscedasticity met (p>0.05) |

**Comment 6: Effect Sizes**

*Reviewer statement:* "Effect sizes should be reported more consistently. Consider reporting confidence intervals alongside β coefficients in regression tables."

**Response:** We have revised all regression tables to include 95% confidence intervals alongside β coefficients. Additionally, we have added Cohen's f² effect size interpretations for key predictors.

**Revised Table 6 (Now includes 95% CI and effect size language):**

| **Variable** | **β (Model 3)** | **95% CI** | **SE** | **p-value** | **Cohen's f²** |
| --- | --- | --- | --- | --- | --- |
| Age | 0.04 | [-0.08, 0.16] | 0.062 | 0.356 | 0.001 |
| Gender | -0.02 | [-0.14, 0.10] | 0.061 | 0.628 | 0.000 |
| Year of Study | 0.09 | [-0.01, 0.19] | 0.051 | 0.081 | 0.012 |
| Previous Clinical Experience | 0.05 | [-0.07, 0.17] | 0.061 | 0.274 | 0.003 |
| Clinical Placement Location | 0.07 | [-0.01, 0.15] | 0.041 | 0.094 | 0.006 |
| **Supervisory Support** | **0.43** | **[0.29, 0.57]** | **0.071** | **<0.001*** | **0.32** |
| **Professional Self-Efficacy** | **0.24** | **[0.10, 0.38]** | **0.083** | **0.002*** | **0.10** |
|  |  |  |  |  |  |
| **Model R²** | **0.537** |  |  |  |  |
| **Model F** | **F(7,137)=21.45** |  |  |  | **p<0.001*** |
| **Overall Effect Size (f²)** |  |  |  |  | **1.16 (Large)** |

*Note: 95% CI = 95% confidence interval; SE = standard error; Cohen's f² interpretation: 0.02 (small), 0.15 (medium), 0.35 (large). Supervisory Support and Professional Self-Efficacy effect sizes are medium to large.*

**Added interpretive text in Results section (Page 20):**

"Effect sizes for key predictors were notable: Supervisory Support demonstrated a medium-to-large effect (Cohen's f²=0.32), while Professional Self-Efficacy demonstrated a small-to-medium effect (Cohen's f²=0.10). The overall regression model showed a large effect size (f²=1.16), explaining 53.7% of the variance in student satisfaction."

**Comment 7: Discussion Length and Repetition**

*Reviewer statement:* "The Discussion section is somewhat lengthy and could be streamlined to reduce repetition, particularly regarding social cognitive theory."

**Response:** We have carefully condensed the Discussion section while retaining all substantive findings and theoretical insights. Specific streamlining efforts:

- Reduced repetition of social cognitive theory: Originally mentioned 4 times throughout Discussion; now consolidated to 2 focused mentions
- Consolidated similar content: Merged multiple paragraphs discussing supervisory support into single, coherent sections
- Eliminated redundancy: Removed repeated statements about "importance of supervision"
- Streamlined "Implications" section: Reduced from 6 paragraphs to 4 focused, distinct implications
- Maintained rigor: All key findings, theoretical connections, and practical recommendations retained

**Comment 8: Typographical and Reference Issues**

*Reviewer statement:* "Repetition: 'effectiveness effectiveness'; Minor punctuation inconsistencies with semicolons; Occasional spacing issues around citations; Some references appear duplicated in citation numbering (e.g., [23] used multiple times). Please verify reference accuracy and numbering."

**Response:** All typographical and reference errors have been systematically corrected:

**REVIEWER #2 RESPONSES**

**Comment 1: Scale Translation and Adaptation Process Documentation**

*Reviewer statement:* "The scales were not translated into Arabic or no description of the translation process. Did you conduct a pilot study to assess if the tool fitting the Saudi Context. Were these scales used in another context?"

**Response:** We have thoroughly documented translation and adaptation (see Reviewer #1, Comment 2, which addresses all of these concerns in detail). Additionally:

**For Supervisory Support Scale:**

- Previously used in workplace settings across multiple countries (US, Europe, Asia)
- Original validation: Cronbach's α=0.93 [Reference 24]
- For this study: Forward-backward translated to Arabic with pilot testing (n=30)
- CFA confirmed factor structure in Saudi context (CFI=0.92, RMSEA=0.064)
- Current study internal consistency: α=0.90

**For Nursing Student Satisfaction Scale:**

- Originally developed for nursing education assessment [Reference 20]
- Adaptation**:** Translated to Arabic and culturally adapted for Saudi nursing students
- Pilot tested with 30 Saudi nursing students
- CFA demonstrated excellent fit (CFI=0.91, RMSEA=0.068)
- Current study reliability: α=0.88

**Comment 2: Data Collection Responsibility and Teaching Involvement**

*Reviewer statement:* "Were you involved in teaching students? Please clarify who was responsible for data collection and what was the response rate?"

**Response:** We have clarified all authorship roles, potential conflicts of interest, and data collection procedures.

**New Text Added to Methods - "Data Collection Procedure" (Page 11):**

"**Data Collection Team and Conflict of Interest Mitigation**

Data collection was conducted by trained research assistants who had no direct teaching responsibilities with participating students at the time of data collection, thereby minimizing potential coercion or demand characteristics in participation decisions. The research team (Dr. Abdulaziz M. Alodhailah, lead supervisor; Drs. Thurayya Eid and Faihan F. Alshaibany) did not directly administer questionnaires but provided supervision and oversight of data collection procedures. This separation of roles between data collectors and academic staff ensured participant autonomy, confidentiality, and reduced social desirability bias.

All team members involved in any aspect of data collection received formal training in research ethics, informed consent procedures, data confidentiality, and participant protection protocols prior to beginning recruitment and data collection activities."

**Updated Authors' Contributions Section (Page 31):**

"**Authors' Contributions**

Conceptualization and Study Design: Thurayya Eid, Faihan F. Alshaibany, Abdulaziz M. Alodhailah
Data Collection: Conducted by trained research assistants under the supervision of Abdulaziz M. Alodhailah. Direct questionnaire administration was conducted by research assistants to minimize coercion or bias related to teaching relationships.
Formal Data Analysis: Thurayya Eid, Faihan F. Alshaibany, Waleed M. Alshehri
Writing – Original Draft: Thurayya Eid, Faihan F. Alshaibany
Writing – Review & Editing, Research Supervision: Abdulaziz M. Alodhailah, Waleed M. Alshehri
Final Approval: All authors read, critically reviewed, and approved the final manuscript."

**Response rate:** Documented above in Reviewer #1, Comment 4 (58%; 145/250 students).

**Comment 3: Professional Self-Efficacy Scale – Source, Item Development, and Validation**

*Reviewer statement:* "You used the professional self-efficacy scale and mentioned that 'Short form adapted from existing instruments'. However, original source unclear, item development process unclear, no content validation."

**Response:** We have now provided comprehensive documentation of the Professional Self-Efficacy Scale development and validation.

**Substantially Expanded Methods Section - Professional Self-Efficacy Scale (Page 17-18):**

**Professional Self-Efficacy Scale: Development, Item Selection, and Validation**

**Professional Self-Efficacy Measurement**
Professional self-efficacy was assessed to evaluate nursing students’ confidence in performing core clinical and professional tasks. A 12-item scale was systematically developed, adapted from established instruments including the Clinical Self-Efficacy Scale [15, 40, 41] and the Nursing Self-Efficacy Scale [42].

**Scale Development Process**

6. **Literature Review and Instrument Analysis:** Key dimensions of professional competence for Saudi undergraduate nursing students were identified through comprehensive review of nursing self-efficacy literature, clinical competency frameworks, and validated instruments.
7. **Expert Consultation Panel:** Eight experts (5 nursing educators, 3 clinical specialists) prioritized core clinical competencies relevant to Saudi healthcare settings.
8. **Item Selection and Dimensionalization:** Twelve items were organized into four empirically-supported dimensions:
   1. Basic and Complex Nursing Procedures (3 items)
   2. Patient and Family Communication (2 items)
   3. Healthcare Team Collaboration and Teamwork (2 items)
   4. Clinical Decision-Making and Prioritization of Care (3 items)
9. **Content Validity Assessment:** An independent panel of 8 nursing experts rated each item on a 4-point relevance scale. All items achieved a CVI ≥ 0.78, with a scale-level CVI of 0.84, indicating excellent content validity.
10. **Cognitive Interviews and Pilot Testing:** The draft scale was pilot-tested with 30 nursing students to assess language clarity, cultural relevance, absence of ambiguity, and response time. Minor wording refinements were made without altering construct meaning.
11. **Construct Validity:** Confirmatory factor analysis on the full sample (n=145) confirmed the four-factor structure (CFI=0.93, TLI=0.91, RMSEA=0.058), with all factor loadings >0.60, supporting construct validity.
12. .

**Scale Administration and Scoring:**
Items are rated on a 5-point Likert scale (1=strongly disagree, 5=strongly agree). Responses are summed to create a total professional self-efficacy score (range: 12–60) and four subscale scores for each dimension. Higher mean scores indicate greater professional self-efficacy. In the current sample, the adapted professional self-efficacy scale demonstrated high internal consistency (Cronbach's α=0.90), with subscale alphas ranging from 0.78–0.85."

**New Table in Appendix A:**

**Table A1. Confirmatory Factor Analysis Results – Professional Self-Efficacy Scale**

| **Dimension** | **Number of Items** | **Factor Loadings (range)** | **Dimension α** |
| --- | --- | --- | --- |
| Basic and Complex Nursing Procedures | 3 | 0.68–0.81 | 0.82 |
| Patient and Family Communication | 2 | 0.72–0.78 | 0.78 |
| Healthcare Team Collaboration | 2 | 0.75–0.83 | 0.80 |
| Clinical Decision-Making | 3 | 0.71–0.79 | 0.83 |
| **Overall Scale (12 items)** | **12** | **0.62–0.83** | **0.90** |

*Note: All factor loadings > 0.60 (excellent); Cronbach's α > 0.78 (very good); CFA fit: CFI=0.93, RMSEA=0.058 (excellent)*

**CONCLUSION**

We have comprehensively and rigorously addressed all reviewer comments and journal requirements. The manuscript now includes:

1. Formal mediation analysis using appropriate bootstrap methods with clear interpretation
2. Complete documentation of instrument adaptation including translation, pilot testing, and construct validity evidence
3. Transparent reporting of methodological decisions including response rate, recruitment, and assumption testing
4. Enhanced statistical reporting with confidence intervals and effect sizes
5. Clarified funding and data availability statements
6. Corrected typographical errors and properly formatted references
7. Streamlined Discussion section maintaining substance while improving conciseness

We believe that the revised manuscript now meets PLOS ONE's high standards for methodological rigor, transparency, and clarity. We appreciate the constructive feedback and are confident that these revisions have substantially strengthened the work.

Sincerely,

Dr. Abdulaziz M. Alodhailah
