# Supplementary material for: Exploring the relationship between supervisory support, self-efficacy, and satisfaction among nursing students in Saudi Arabia
Source: PLoS One. 2026 Jul 16;21(7):e0352318. doi: 10.1371/journal.pone.0352318 (PMC13375050; doi:10.1371/journal.pone.0352318)
Supplement: Attachment — Submitted filename: Response_to_Reviewers_Letter_auresp_2.docx [file pone.0352318.s005.docx]

**Response to Reviewers**

Manuscript ID: PONE-D-26-01430R1
Title: Exploring the Relationship Between Supervisory Support, Self-Efficacy, and Satisfaction Among Nursing Students in Saudi Arabia

Authors: Thurayya Eid, Faihan F. Alshaibany, Abdulaziz M. Alodhailah, Waleed M. Alshehri

Dear Dr. Alamri and Reviewers,

We would like to sincerely thank you and the reviewers for the careful evaluation of our manuscript and for the constructive and encouraging feedback. We are pleased that the manuscript has been found to be substantially improved, technically sound, and suitable for publication pending minor revisions.

We have carefully addressed all remaining comments. All changes have been incorporated into the revised manuscript and are highlighted in the tracked changes version. Below, we provide a detailed, point-by-point response.

### **Reviewer #1**

**Comment 1: Typographical Error - Duplicated Word**

A minor typographical issue remains in the introduction (“effectiveness effectiveness”), which should be corrected.

**Response:**
Thank you for identifying this typographical error. We have corrected the duplicated word in the Introduction section.

**Comment 3: Statistical Reporting — Power Analysis Effect Size**
The power analysis section refers to Cohen’s d (0.3). Since the study uses regression models, it may be more appropriate to report effect size in terms of f².

**Response:**
We have revised the power analysis section to report Cohen's f² instead of Cohen's d. Following the conversion formula $f^2 = \frac{d^2}{4}$ (Cohen, 1988, p. 276; Selya et al., 2012), the originally specified Cohen's d = 0.3 (medium effect) corresponds to:

$$f^2 = \frac{0.3^2}{4} = \frac{0.09}{4} = 0.0225$$

Change Made (Methods, Sample and Sampling section, page 6–7)

However, this would be a very small effect size for regression (below the f² = 0.02 "small" threshold is near, and the resulting sample would be very large). Given that we planned for a medium effect in regression, and our actual results confirmed medium-to-large effects (f² = 0.32 for supervisory support; overall model f² = 1.16), we have revised the power analysis to directly specify a medium effect size of f² = 0.15 (Cohen, 1988), which is standard for multiple regression. Using G*Power for linear multiple regression with f² = 0.15, α = 0.05, power = 0.80, and 7 predictors, the required minimum sample size is approximately 103. Our achieved sample of 145 exceeds this threshold and provides adequate statistical power.

**Comment 3:**
Given the cross-sectional design, I suggest slightly softening the interpretation of mediation to emphasize statistical mediation rather than causal inference.

**Response:**
Thank you for this insightful comment. We agree with this recommendation and have revised the relevant sections of the manuscript (Abstract, Results, and Discussion) to clarify that the mediation analysis reflects **statistical associations rather than causal relationships**. The language has been softened to avoid causal interpretations and to align with the limitations of a cross-sectional design.

### **Reviewer #2**

**Comment:**
Thank you for addressing all of my comments in the revised manuscript. I do not have any further comments.

**Response:**
We sincerely thank the reviewer for their positive feedback and for their valuable input throughout the review process, which has helped strengthen the quality of our manuscript.

### **Additional Revisions**

- We have carefully reviewed the entire manuscript to ensure clarity, consistency, and accuracy.
- Minor language edits have been made to improve readability.

We hope that the revised manuscript meets the journal’s requirements and is now suitable for publication in PLOS ONE. We appreciate your time and consideration.

Kind regards,
Dr. Abdulaziz M. Alodhailah
On behalf of all authors
King Saud University
Email: [aalodhailah@ksu.edu.sa](mailto:aalodhailah@ksu.edu.sa)
