# Supplementary material for: Exploring the relationship between supervisory support, self-efficacy, and satisfaction among nursing students in Saudi Arabia
Source: PLoS One. 2026 Jul 16;21(7):e0352318. doi: 10.1371/journal.pone.0352318 (PMC13375050; doi:10.1371/journal.pone.0352318)
Supplement: Attachment — Submitted filename: Response_to_Reviewers.docx [file pone.0352318.s006.docx]

POINT-BY-POINT RESPONSE TO REVIEWER COMMENTS

Manuscript Title: Supervisory Support, Professional Self-Efficacy, and Student Satisfaction in Nursing Clinical Education: A Cross-Sectional Study in Saudi Arabia

The authors thank the reviewer for their thorough and constructive feedback. We have addressed each comment systematically below. All revisions are reflected in the tracked-changes manuscript. We believe the manuscript has been substantially strengthened as a result.

ABSTRACT

\#	Reviewer Comment	Authors’ Response

A.1	Please provide a more concise summary of the study background.	Done. The abstract background has been condensed to four sentences that convey the core rationale without restating the introduction in detail.

A.2	The general objective of the study should be explicitly stated at the end of the background section.	The revised abstract background now concludes with an explicit statement of the study’s aim.

A.3	Clearly state the study design in complete form, e.g., ‘This descriptive cross-sectional study was conducted among 145 nursing students at King Saud University hospitals.’	The Methods sentence now reads: “This descriptive cross-sectional study was conducted among 145 nursing students (second-, third-, and fourth-year bachelor’s degree students) at three hospitals affiliated with King Saud University in Riyadh, Saudi Arabia.”

A.4	‘Descriptive statistics summarized study variables’ is too general. Specify the key study variables.	Revised to: “Descriptive statistics summarised the three key study variables (supervisory support, professional self-efficacy, and student satisfaction) alongside sociodemographic characteristics.”

A.5	‘Pearson correlations examined relationships’ should be revised to reflect the purpose more accurately.	Revised to: “Pearson correlation coefficients assessed the strength and direction of linear associations among continuous study variables.”

A.6	Key socio-demographic characteristics should be briefly presented before reporting main results.	Added to abstract Results: “Of 250 eligible students, 145 completed the questionnaire (response rate = 58%). The sample was predominantly female (60.7%) and aged 20–22 years (66.9%).”

A.7	Provide the response rate at the beginning of the Results section.	Response rate (58%, 145/250) is now the opening statement of the abstract Results section.

A.8	Clarify what reported mean scores are based on (e.g., ‘out of 5’) and ensure consistent reporting of means with SD.	All means in the abstract are now reported as “mean = X.XX ± SD out of 5.”

A.9	Key regression results should be clearly and concisely stated.	The abstract now states: “In regression analysis, supervisory support remained significantly associated with satisfaction (β = 0.43, p < 0.001) even after accounting for professional self-efficacy, consistent with partial statistical mediation.”

BACKGROUND

\#	Reviewer Comment	Authors’ Response

B.1	Include a dedicated paragraph discussing key challenges and barriers related to supervisory support and professional self-efficacy in nursing education.	A new paragraph has been added to the Background detailing challenges including high instructor workloads, variability in supervision quality, limited feedback opportunities, and student–supervisor communication gaps, with supporting citations [3, 5, 21].

B.2	‘The quality of supervisory support is paramount’ should be strengthened with concrete examples of how supervisory support influences outcomes.	The relevant sentence now cites specific outcomes: clinical skill development, professional identity formation, and transition to practice readiness.

B.3	Several paragraphs contain grammatical and stylistic issues.	The entire Background section has been revised for grammatical accuracy, formal academic register, and stylistic consistency throughout.

B.4	The role of clinical instructors should be elaborated to highlight direct influence on learning outcomes and clinical competence.	Revised to: “…clinical instructors… play a direct role in shaping clinical competence, professional identity, and readiness for independent practice [9].”

B.5	The hypothesis statement should be clearly positioned immediately before the objectives.	The hypothesis sentence (“Guided by social cognitive theory, we hypothesised…”) now immediately precedes the study objectives at the end of the Background.

B.6	Clearly explain how Social Cognitive Theory guided variable selection and how constructs were operationalised.	A new sentence now explains that supervisory feedback operationalises verbal persuasion, practitioner observation operationalises vicarious learning, and graduated practice operationalises mastery experience, per Bandura [19].

B.7	A dedicated paragraph should be added discussing the potential impact of the study on clinical practice, education, management, and research.	Added. The new paragraph addresses: clinical practice (supervision quality and patient care), education (curriculum and student support), management (supervisor training and placement organisation), and research (future interventional and longitudinal studies).

METHODS

\#	Reviewer Comment	Authors’ Response

M.1	Summarize the study design section more concisely while maintaining essential details; align with STROBE.	Revised to one concise paragraph: “This descriptive cross-sectional study examined associations among supervisory support, professional self-efficacy, and nursing student satisfaction. Reporting adheres to STROBE guidelines.”

M.2	Specify the number and names of the hospitals affiliated with King Saud University and justify their selection.	The Setting section now names all three hospitals (KSUMC, KKUH, KAUH) and provides explicit justification (accessibility, structured training programmes, representativeness of the KSU system).

M.3	The rationale for a single-university setting should be stated; this is a limitation.	The rationale is noted in Methods (representativeness of KSU clinical system) and the limitation of single-university generalisability is explicitly acknowledged in the Strengths and Limitations section.

M.4	Specify the assumed attrition rate and avoid repetition of the non-response explanation.	Revised to specify an estimated 10–15% non-response rate as the basis for increasing the target to 145. The duplicated sentence has been removed.

M.5	Report the total target population size with a reference; describe the formula for sample size estimation.	Population size (approximately 250 students enrolled during the study period) is reported. G*Power 3.1 and the parameters for a priori power analysis are now explicitly described with citation [44].

M.6	Clarify the stratified random sampling procedure, including strata definition and allocation.	The Methods now describes stratification by year of study (second, third, fourth year) and states that proportionate allocation was used to ensure representation at each stage.

M.7	‘Students who had completed at least one clinical rotation’ — clarify terminology.	Revised to: “having completed at least one supervised clinical placement in a hospital department under the university curriculum.”

M.8	Inclusion criteria should include academic level and educational level.	Inclusion criteria now explicitly state: “enrolled in the second, third, or fourth year of the KSU bachelor’s degree nursing programme.”

M.9	Specify the exact nature of the data collection tools.	The Methods now states that instruments were structured self-administered questionnaires administered in paper form.

M.10	The tool selection rationale is too general; explain psychometric properties more clearly.	The Data Collection Instruments section now explains how Cronbach’s α, CFA fit indices, and prior validation evidence informed instrument selection.

M.11	The sentence explaining subgroup analysis context is not essential and should be removed.	Removed.

M.12	Provide a detailed description of the translation/adaptation process, number and expertise of experts, and modifications made. Also clarify why construct validity was assessed after data collection.	The adaptation process is now described in detail: forward translation by two bilingual nursing education experts, backward translation by two independent translators, consensus review, pilot testing with n = 30 students. The justification for post-collection CFA (full sample required for adequate CFA power) is noted.

M.13	Explain how levels (high/moderate/low) were determined.	Now specified: trisection of the 1–5 Likert scale, with low ≤ 2.33, moderate 2.34–3.66, high ≥ 3.67, consistent with published scoring conventions [24].

M.14	Data collection procedures are described more than once; merge under a single subheading.	All data collection content consolidated under a single “Data Collection Procedure” subheading.

M.15	Non-response bias statement requires clarification.	Clarified: χ² comparisons of age group, gender, and year of study between respondents and non-respondents using institutional enrolment records are now reported with test statistics and p-values.

M.16	Recalculate and clearly report response rate based on completed questionnaires / sampled participants.	Response rate = 145/250 = 58% is now the first sentence of the non-response bias paragraph.

M.17	Data collection sentence should use formal academic language.	Revised: “Questionnaires were administered face-to-face at the three participating hospitals by trained research assistants.”

M.18	Statistical analysis section should be summarised into one coherent paragraph.	Consolidated into a single paragraph covering all statistical methods, significance thresholds, and software.

M.19	Correlation analysis statement is not accurate — Pearson should be limited to continuous variables.	Revised. The Methods now states that Pearson correlation assessed linear associations among continuous variables only, and notes that the binary gender variable’s correlation with continuous outcomes should be interpreted cautiously.

RESULTS

\#	Reviewer Comment	Authors’ Response

R.1	Report whether assumptions for Pearson correlation, regression, and ANOVA were assessed before analysis.	Added to Results opening: “All regression model assumptions were satisfied prior to analysis (see Methods and Appendix B).” The Methods section now contains a full Regression Model Assumptions Testing subsection.

R.2	Clarify rationale for including age and gender in correlation analysis.	Age (continuous) is appropriate for Pearson correlation. Gender (binary) is now flagged in both the correlation table footnote and text as a descriptive-only measure interpreted with caution.

R.3	Write the name of the table at the end of each relevant paragraph.	Each results paragraph now ends with a parenthetical table citation, e.g., (Table 6).

R.4	Table numbering is duplicated (Table 9 appears twice).	Corrected. The ANOVA table is now Table 10; all table numbers are sequential and consistent throughout.

R.5	Provide a concise narrative summary for each table.	Each table is accompanied by 1–2 narrative paragraphs highlighting key findings. Summary language is in the text; tables present the full data.

R.6	Ensure interpretation of correlation coefficients follows accepted guidelines.	Correlation magnitudes are now described using accepted benchmarks (r ≈ 0.50 = moderate, r ≥ 0.60 = strong) consistent with standard conventions.

R.7	Some statements contain interpretation rather than pure reporting (e.g., ‘likely due to increased familiarity’). Move to Discussion.	Interpretive language has been removed from Results and incorporated into the Discussion.

R.8	Mediation findings should be interpreted as statistical mediation, not causal.	Throughout the Results and Discussion, all mediation language is qualified: “statistical mediation,” “statistical association,” and the cross-sectional design limitation is noted explicitly in the table footnote.

R.9	Explain how categorical variables were coded for regression models.	Now specified: “Gender, year of study, and clinical placement location were dummy-coded for entry into regression models.” This appears in the Methods and is noted in Table 6 footnotes.

R.10	Since ANOVA showed significant differences, provide post-hoc comparisons (e.g., Tukey).	Post-hoc Tukey test results are now reported for both year-of-study and clinical placement location comparisons (Table 9 footnotes and Results narrative).

R.11	Verify consistency between text, tables, beta coefficients, p-values, and CIs.	All values have been cross-checked for consistency between narrative text and tables. No discrepancies remain.

R.12	Clarify whether standardised or unstandardised coefficients are reported.	Clarified throughout: β = standardised regression coefficients in all tables and text. This is noted in all table footnotes.

R.13	Findings are repeated multiple times in text and tables. Reduce redundancy.	Redundant restatements have been removed. The text summarises key findings; tables present full data.

R.14	State whether mediation assumptions were assessed and whether PROCESS included covariates.	Added to Results: mediation assumptions are described (paths a, b significant; total effect significant). PROCESS Model 4 is noted; no additional covariates beyond the mediator were included in the PROCESS model.

R.15	Report effect sizes for all major analyses.	Cohen’s f² is reported for all regression models (Table 6). η² is reported for the ANOVA (Table 10 footnote). Correlation coefficients serve as their own effect size metrics.

R.16	Describe missing data and how managed.	Added: “Missing data were negligible: no item-level missing data were observed, as research assistants verified questionnaire completeness at the point of collection.”

R.17	Some tables are overly dense; improve formatting and alignment of decimal places.	Tables have been reformatted with consistent decimal alignment, adequate cell padding, and simplified structure where possible.

R.18	Causal language (influencing, effect, predictors) should be replaced with associative language.	All instances of ‘influence,’ ‘effect,’ and ‘predict’ in the Results have been replaced with ‘associated with,’ ‘explained variance,’ or equivalent associative terminology.

DISCUSSION

\#	Reviewer Comment	Authors’ Response

D.1	Discussion should focus on interpretation, not restatement of statistical values.	The Discussion has been restructured to emphasise theoretical interpretation and contextualisation. Specific statistical values are cited only where necessary for interpretive precision; detailed results remain in the Results section.

D.2	Causal language (influence, predictor, fosters) should be replaced with associative terminology.	All causal language in the Discussion has been replaced with ‘associated with,’ ‘statistically mediated,’ and similar appropriate phrasing.

D.3	Several findings are discussed without sufficient comparison to regional or international studies.	Each thematic subsection now includes direct comparison with international evidence (Singapore [13], US [14]) and acknowledges where findings align with or diverge from prior research.

D.4	Social Cognitive Theory is mentioned but the discussion should critically explain how findings support or extend it.	A new paragraph explicitly explains how study findings instantiate SCT mechanisms: mastery experience, vicarious learning, and verbal persuasion, and how partial mediation extends the theory empirically.

D.5	Discussion repeatedly restates statistical values from Results.	Removed. The Discussion now references Results figures minimally and only where they anchor an interpretive point.

D.6	Cultural, educational, and institutional characteristics in Saudi Arabia should be discussed more clearly.	A dedicated paragraph now addresses hierarchical organisational culture, gender dynamics in clinical settings, and institutional change under Vision 2030 as contextual factors shaping the findings.

D.7	Inter-hospital differences are mentioned briefly; provide more in-depth discussion of contributing factors.	The inter-hospital variation is now discussed in a dedicated subsection covering supervisory practices, resource availability, staffing ratios, and organisational culture as plausible explanatory factors.

D.8	Acknowledge potential biases (self-report, social desirability, unmeasured confounders).	Added to Discussion and Limitations: self-report bias, social desirability effects, common method variance, and unmeasured contextual variables are all explicitly acknowledged.

D.9	Mediation findings should be described as statistical mediation, not causal.	All mediation language in the Discussion uses ‘statistical mediation’ and the cross-sectional design constraint is explicitly noted.

D.10	Implications for educators, supervisors, and policymakers should be expanded with specific recommendations.	The Implications section has been substantially expanded with specific, actionable recommendations linked directly to study findings, including supervisor training, structured feedback, cross-site quality assurance, and interprofessional education.

D.11	Comprehensive acknowledgement of study limitations is needed.	The Strengths and Limitations section now comprehensively addresses: cross-sectional design, single-university setting, self-report bias, common method variance, unmeasured confounders, response bias, and generalisability constraints.

D.12	Some statements are overly conclusive given observational design. Moderate claims.	All claims have been moderated. Language such as ‘demonstrates,’ ‘proves,’ and ‘causes’ has been replaced with ‘suggests,’ ‘is consistent with,’ and ‘is associated with.’

D.13	Non-significant findings (age, gender) should be critically interpreted.	The Discussion now includes a sentence explaining why age and gender may not have been associated with outcomes in this sample: narrow age range (17–22 years), limited variance, and the specific institutional context.

D.14	Discuss other potential factors contributing to the 46.3% unexplained variance.	Added: unmeasured factors including supervisor–student relationship quality, interprofessional team dynamics, clinical environment characteristics, and individual resilience are identified as plausible contributors to unexplained variance.

D.15	Discuss other possible mediating mechanisms beyond professional self-efficacy.	Added: emotional support dimensions of the supervisory relationship, relationship quality, and mentorship are identified as plausible additional mediating pathways consistent with the large direct effect (β = 0.43).

D.16	Discuss feasibility and resource implications of implementing recommendations.	The Implications section now addresses feasibility: cross-site standardisation within a single-university system is specifically noted as achievable given existing administrative relationships.

D.17	Ensure recommendations are directly supported by study findings.	All recommendations are now explicitly anchored to specific findings (e.g., supervisor training linked to the supervisory support coefficient; cross-site standardisation linked to the inter-hospital ANOVA).

D.18	Improve overall flow and synthesis across discussion themes.	The Discussion has been reorganised into clearly delineated thematic subsections with transitional prose that integrates findings across themes.

IMPLICATIONS OF THE STUDY

\#	Reviewer Comment	Authors’ Response

I.1	Ensure educational and practice implications are directly linked to study findings.	All five implications are now anchored to specific results: supervisor training (linked to β = 0.52/0.43), self-efficacy development (linked to mediation analysis), cross-site standardisation (linked to ANOVA), teamwork (linked to lowest satisfaction item), and Vision 2030 (linked to overall findings in the Saudi context).

I.2	Recommendations should be framed more cautiously given cross-sectional design.	Recommendations are now framed as “priority strategies” and “potential interventions” rather than confirmed solutions, with appropriate hedging language throughout.

I.3	Add specific examples of how programmes can implement supervision and self-efficacy strategies.	Specific mechanisms are now proposed: competency-based milestone recognition, structured simulation before complex tasks, peer learning, supervisor communities of practice, and shared outcome benchmarking.

I.4	Discuss feasibility and challenges of supervisor training and quality assurance.	Feasibility is now explicitly addressed: within-university cross-site standardisation is noted as achievable; recommendations are proportionate to available institutional infrastructure.

I.5	The Vision 2030 connection needs more specific explanation.	Expanded: the implications section now explains how improved supervision and self-efficacy development directly support Saudi Arabia’s goals of increasing locally trained nurses and improving healthcare quality under Vision 2030.

STRENGTHS AND LIMITATIONS

\#	Reviewer Comment	Authors’ Response

S.1	Emphasize that single-university setting limits external generalizability.	Explicitly stated: “Conducting the study within a single university system in Riyadh limits external generalisability to other institutions, regions, or countries.”

S.2	Common method variance from single-time-point self-report should be acknowledged.	Added: “All variables were measured using self-report questionnaires at a single time point, introducing potential common method variance, social desirability bias, and recall bias.”

S.3	Clarify whether full measurement invariance or cross-cultural validation was tested.	Clarified: CFA confirmed local construct validity; full measurement invariance and cross-cultural validation beyond the Saudi context have not been established. This is noted as a limitation.

S.4	High mean scores may indicate response bias; discuss explicitly.	Added: “The relatively high mean scores may reflect a positively biased sample or ceiling effects on instruments.” The limitations section discusses this alongside the non-response bias assessment.

CONCLUSION

\#	Reviewer Comment	Authors’ Response

C.1	Conclusion restates findings rather than synthesising and highlighting contribution.	Revised to focus on synthesis and contribution: “This study provides the first empirically grounded mediation model of the supervisory support–self-efficacy–satisfaction pathway within Saudi Arabia’s teaching hospital system.”

C.2	‘Supervisory support may enhance learning’ implies causality.	Revised to: “Supervisory support is associated with students’ satisfaction…” Causal language removed throughout.

C.3	Include a clearer statement of the study’s unique contribution.	Added explicitly: the study’s unique contribution is characterised as the first mediation model of these relationships in Saudi Arabia’s clinical education system at a moment of national healthcare transformation.

C.4	Condense and prioritise the most critical practice implications.	The conclusion’s recommendations paragraph has been condensed to three actionable priorities, with less critical specifics moved to the Implications section.

C.5	Suggest specific future directions beyond longitudinal studies.	Future directions now include: longitudinal designs, multi-site studies across Saudi institutions, qualitative exploration of supervisory practices, and interventional studies testing structured supervision programmes.

We are grateful for the thoroughness and rigour of the reviewer’s comments. All requested revisions have been incorporated. We are confident that the revised manuscript meets the standards expected by PLOS ONE and makes a meaningful contribution to the nursing education literature. We welcome any further feedback.

Respectfully submitted on behalf of all authors,

Dr. Abdulaziz M. Alodhailah

College of Nursing, King Saud University, Riyadh, Saudi Arabia

On behalf of: Dr. Thurayya Eid, Dr. Faihan F. Alshaibany, Dr. Waleed M. Alshehri
