# Supplementary material for: Exploring the relationship between supervisory support, self-efficacy, and satisfaction among nursing students in Saudi Arabia
Source: PLoS One. 2026 Jul 16;21(7):e0352318. doi: 10.1371/journal.pone.0352318 (PMC13375050; doi:10.1371/journal.pone.0352318)
Supplement: Attachment — Submitted filename: Response_to_Reviewers_auresp_4.docx [file pone.0352318.s007.docx]

Response to Reviewers

Manuscript ID: PONE-D-26-01430R3

Dear Dr. Majed Alamri and Members of the Editorial Board,

We sincerely thank the Editor and all three Reviewers for their careful reading of our revised manuscript entitled "Exploring the Relationship Between Supervisory Support, Self-Efficacy, and Satisfaction Among Nursing Students in Saudi Arabia." We have thoroughly revised the manuscript in response to every point raised. Below we provide a point-by-point account of all changes made. All revisions are indicated as tracked changes attributed to Dr. Abdulaziz Alodhailah in the accompanying Revised Manuscript with Track Changes file.

Reviewer #1

Comment 1.1

Thank you for the revised manuscript. The authors have addressed the majority of the previous comments thoroughly, and the manuscript has improved substantially in methodological clarity, theoretical integration, statistical reporting, and overall presentation. All comments have been addressed.

Response:

We thank Reviewer #1 for this positive assessment. The current revision further addresses all remaining points raised by Reviewer #6, as detailed below.

Reviewer #4

Comment 4.1

Thank you for providing further revision on this paper. Note that I have not reviewed any statistical analysis associated with your study. All comments have been addressed.

Response:

We thank Reviewer #4. Statistical concerns raised in this round by Reviewer #6 have been fully addressed, including re-analysis of group comparisons in Table 9 using appropriate tests (see Comment 6.10 below).

Reviewer #6

General Comment

The authors are commended for their efforts. However, several issues remain that require further major revision to improve clarity, methodological rigour, statistical reporting, and academic writing quality. The Discussion section requires substantial revision and a comprehensive rewrite, with a stronger emphasis on detailed comparison with relevant previous studies. The manuscript contains several grammatical and linguistic errors.

Response:

We thank Reviewer #6 for this thorough critique. We have systematically revised every section of the manuscript in response. The Discussion has been substantially expanded with direct comparisons to prior studies, and grammatical clarity has been improved throughout. Each specific point is addressed below.

Comment 6.1 - Abstract: Hospital Names

It is not necessary to include the names of the hospitals in the abstract. Please remove them from the abstract.

Response:

We agree. The three hospital names have been removed from the Abstract Methods sentence. The sites are now described collectively as three King Saud University-affiliated teaching hospitals in Riyadh, Saudi Arabia, which preserves the geographic and institutional context without listing individual names.

Change in manuscript:

Abstract, Methods sentence: removed '(King Saud University Medical City, King Khalid University Hospital, and King Abdulaziz University Hospital)'. Now reads: 'three King Saud University-affiliated teaching hospitals in Riyadh, Saudi Arabia'.

Comment 6.2 - Abstract: Response Rate Calculation

The response rate calculation requires revision. It should be clearly based on the appropriate sampling frame rather than the total population. Please recheck the calculation and ensure consistency between the sample size determination and the reported response rate.

Response:

We agree the denominator was inappropriate. The previous figure of 58% divided final participants (145) by total enrolled students (250), not by the number approached. The 175 students simultaneously on clinical placement at the three hospitals during the study period constitute the correct sampling frame (based on institutional scheduling records), giving a response rate of 82.9% (145/175). This figure is now used consistently across the Abstract, Methods, and Results.

Change in manuscript:

Abstract Results: 'Of 250 eligible students, 145 completed the questionnaire (response rate = 58%)' corrected to 'Of 175 students approached and meeting eligibility criteria, 145 returned valid questionnaires (response rate = 82.9%)'. Same correction applied in Methods (Sample Size paragraph) and Results (opening sentence).

Comment 6.3 - Background: Missing Citation (Effective Supervision)

Please add a supporting citation for the following statement: 'Effective supervision creates environments where students develop critical thinking, clinical decision-making, and hands-on nursing skills.'

Response:

A citation has been added. We cite Javornicka et al. [3], Zhang et al. [5], and Laugaland et al. [46], all of which directly document associations between supervisory quality and development of clinical reasoning and practical nursing skills.

Change in manuscript:

Background, paragraph 1: Added '[3, 5, 46]' after 'hands-on nursing skills.' Reference [46] (Laugaland et al., BMC Nurs., 2021) added to reference list.

Comment 6.4 - Background: Missing Citations (Supervision Challenges)

Provide appropriate citations for the following statements: 'Despite broad consensus on the value of supervision, significant challenges persist. Clinical instructors often face high workloads and variable caseloads that limit individualised student contact. Supervision quality varies considerably across sites owing to differences in instructor experience, institutional resources, and organisational culture.'

Response:

Citations have been added to each claim. Workload and caseload constraints are now cited with Stevenson et al. [47] and Jokelainen et al. [48]. Site-level variation in supervision quality is cited with Hilli et al. [49] and Chuan and Barnett [50]. All four references have been added to the reference list.

Change in manuscript:

Background, paragraph 2: '[47, 48]' inserted after 'individualised student contact'; '[49, 50]' inserted after 'organisational culture'. References [47]-[50] added to reference list.

Comment 6.5 - Background: Self-Efficacy Mechanism Statement

The authors need to clarify the following statement by specifying the mechanisms and adding appropriate citations: 'In the clinical education context, these mechanisms are operationalised through supervisor feedback (verbal persuasion), direct observation of skilled practitioners (vicarious learning), and graduated practice under supportive conditions (mastery experience).'

Response:

The sentence has been substantially revised. Bandura's four sources of self-efficacy are now named explicitly and in the order he presents them, each linked to a specific clinical education activity, with the applied nursing literature cited alongside Bandura [19]. The revision cites Chesser-Smyth [51], whose phenomenological study directly maps Bandura's mechanisms to student nurse clinical placement experiences.

Change in manuscript:

Background, paragraph 4: Original sentence replaced with: 'In the clinical education context, Bandura's [19] four sources of self-efficacy are operationalised as follows: mastery experience, through supervised clinical tasks of progressively increasing complexity; vicarious learning, through observation of competent instructors and peers performing clinical skills; verbal persuasion, through constructive, specific feedback delivered by supervisors; and management of physiological states, through strategies that reduce anxiety during clinical performance [15, 51].' Reference [51] (Chesser-Smyth, 2005) added to reference list.

Comment 6.6 - Methods: Sample Size and Response Rate Consistency

The sample size is reported as 103 based on calculation and later increased to 145 participants. However, the reported response rate of 58% requires clarification and revision. Please ensure consistency between the sample size calculation, population size, and final included participants.

Response:

This has been clarified in the Methods section. The minimum required sample (n = 103) was derived from the power analysis. The recruitment target was set at 175 - the number of eligible students simultaneously on clinical placement at the three sites - to ensure precision beyond the minimum. Of these, 145 returned valid questionnaires (response rate = 82.9%). The manuscript now clearly distinguishes: (a) power-analysis minimum (103); (b) recruitment target/sampling frame (175); (c) final sample (145; response rate 82.9%).

Change in manuscript:

Methods, Sample Size paragraph: 'To account for an estimated 10-15% non-response rate, the recruitment target was set at 145 participants...' replaced with explanation of 175-student sampling frame, 145 completions, and 82.9% response rate, with institutional source cited.

Comment 6.7 - Methods: Population Size Must Be Referenced

The total population size (250 students) must be properly referenced.

Response:

The figure is now attributed to institutional scheduling records (King Saud University College of Nursing, personal communication, 2024), cited in the revised Methods paragraph in accordance with PLOS ONE reference guidelines for personal communications.

Change in manuscript:

Methods, Sample Size paragraph: Institutional source for the 175-student sampling frame explicitly cited as 'King Saud University College of Nursing, personal communication, 2024'.

Comment 6.8 - Methods: Remove Non-Response Bias Paragraph

I suggest you remove the attached paragraph from the methods section: 'Response rate and non-response bias. Of approximately 250 students enrolled... suggesting the final sample was reasonably representative of the broader population.'

Response:

This paragraph has been deleted in full. As the reviewer correctly notes, because a purposive sampling frame was used, comparing respondents to the total enrolled population is methodologically inconsistent with the design. The deletion is marked as a tracked change in the manuscript.

Change in manuscript:

Methods, Data Collection Procedure section: Paragraph headed 'Response rate and non-response bias' (including chi-square comparisons) deleted in full.

Comment 6.9 - Methods: Revise Response Rate Sentence

The following statement should be revised for clarity and accuracy: 'Of approximately 250 students enrolled in clinical placements at the three hospitals during the study period, 145 returned valid questionnaires (response rate = 58%).'

Response:

This sentence has been deleted as part of the removal of the non-response bias paragraph (Comment 6.8). The corrected response rate (82.9%) now appears in the revised Sample Size paragraph and in the opening sentence of the Results.

Change in manuscript:

See Comments 6.6 and 6.8. Results opening sentence now reads: 'Of 175 students approached and meeting eligibility criteria, 145 returned valid questionnaires (response rate = 82.9%).

Comment 6.10 - Results: Inappropriate ANOVA for Gender in Table 9

In Table 9, ANOVA p-values are reported for variables where ANOVA is not appropriate for several variables such as gender. Please reanalyse the data using appropriate statistical tests.

Response:

We thank the reviewer for identifying this error. For the dichotomous variable gender (male vs. female), ANOVA has been replaced with an independent samples t-test. For variables with three or more categories (year of study; clinical placement location), one-way ANOVA with post-hoc Tukey tests remains appropriate and is retained. The Methods (Statistical Analysis), Table 9 caption, the Table 9 column heading, the Table 9 footnote, and the Results text for Table 9 have all been updated accordingly. The gender p-value in Table 9 now reflects the t-test result (p = 0.318).

Change in manuscript:

Methods: New sentence specifying t-test for dichotomous variables and ANOVA for 3+ category variables. Table 9 caption: 'ANOVA Results' -> 'Statistical Test Results'. Column header: 'p-value (ANOVA)' -> 'p-value (statistical test)'. Gender p-value: 0.373 -> 0.318 with dagger footnote: 'Independent samples t-test used for gender (dichotomous variable); one-way ANOVA used for all other comparisons.' Results text updated to specify test type for each variable.

Comment 6.11 - Results: Response Rate in Opening Sentence

The authors should write the accurate response rate in the first sentence in the results section.

Response:

The opening of the Results has been revised. The cross-reference 'see Methods and and Appendix B' (which also contained a typographic double-word error) has been corrected to 'see Appendix B', and a new sentence has been inserted: 'Of 175 students approached and meeting eligibility criteria, 145 returned valid questionnaires (response rate = 82.9%).'

Change in manuscript:

Results, opening paragraph: Added sentence with corrected response rate (82.9%). Typographic error 'and and' corrected to 'and'.

Comment 6.12 - Results: Table References at End of Paragraphs

The authors must write the number of several tables at the end of the paragraphs, not in the first sentence, such as 'Table 1 presents the sociodemographic characteristics of the sample (n = 145).'

Response:

All Results paragraphs have been reviewed. Where a table number appeared as the grammatical subject of the opening sentence, it has been moved to a parenthetical citation at the end of the paragraph. The example given by the reviewer (Table 1) has been corrected, and equivalent instances elsewhere have been revised.

Change in manuscript:

Results, Sociodemographic Characteristics paragraph: 'Table 1 presents the sociodemographic characteristics of the sample (n = 145). The majority...' -> 'The majority... (Table 1).' Equivalent restructuring applied throughout Results section.

Comment 6.13 - Discussion: Substantial Revision Required

This section requires substantial revision, as its current quality is insufficient for publication standards. The discussion should be rewritten in a more coherent, critical, and evidence-based academic style.

Response:

The Discussion has been substantially revised throughout. Each subsection now: (1) opens with the empirical finding; (2) compares it explicitly with a named prior study using specific values where available; and (3) closes with a theoretically grounded interpretation tied to social cognitive theory. New comparative literature has been incorporated across all subsections (see Comment 6.15 for detail).

Change in manuscript:

Discussion, all subsections: New comparative sentences inserted citing Javornická et al. [3], Woo and Li [13], Phillips et al. [14], Khari et al. [15], Abuadas [20], Donough and Van der Heever [21], Zhang et al. [5], Martin et al. [4], Burgess et al. [39], and Laugaland et al. [2].

Comment 6.14 - Discussion: Avoid Overemphasis on Numerical Values

Avoid overemphasis on numerical values within the discussion section such as 'The significant variation in satisfaction across the three hospitals (ANOVA: F = 4.312, p = 0.016; eta-squared = 0.058) warrants particular attention'.

Response:

This sentence has been revised to: 'Satisfaction differed significantly across the three hospital sites, with a small-to-medium effect size.' The F-value, p-value, and effect size have been removed from the Discussion prose. We applied this principle consistently: all F-values, beta coefficients, and exact p-values that appeared in the Discussion prose have been converted to plain-language descriptions of significance and direction, with readers directed to the Results tables for full statistics.

Change in manuscript:

Discussion, Institutional and Cultural Context subsection: '...The significant variation in satisfaction across the three hospitals (ANOVA: F = 4.312, p = 0.016; eta-squared = 0.058) warrants particular attention.' -> 'Satisfaction differed significantly across the three hospital sites, with a small-to-medium effect size.'

Comment 6.15 - Discussion: Strengthen Comparison with Prior Studies

Several subsections lack adequate comparison with existing literature. Please strengthen the discussion by: (1) comparing findings with relevant previous studies; (2) providing scientific justification for key results; (3) strengthening theoretical and empirical interpretation.

Response:

Each subsection of the Discussion has been augmented with direct empirical comparisons:

(1) Levels paragraph: Abuadas [20] cited to contextualise high scores within Saudi teaching hospitals.

(2) Feedback paragraph: Burgess et al. [39] systematic review cited to justify why feedback was the most valued supervisory item.

(3) Self-efficacy paragraph: Khari et al. [15] cited for direct comparison of self-efficacy domain profiles (communication and teamwork high; complex procedures and prioritisation lower in both studies).

(4) Associations paragraph: Javornická et al. [3] (r = 0.65) and Woo and Li [13] cited to contextualise r = 0.68; Phillips et al. [14] (r = 0.46) cited for the self-efficacy-satisfaction association.

(5) Predictors paragraph: Zhang et al. [5] and Martin et al. [4] cited for the dominance of supervisory support as a predictor; Khari et al. [15] cited for the comparable 22% mediation proportion.

(6) Hospital variation paragraph: Donough and Van der Heever [21] cited for structural drivers of site-level differences.

(7) Year of study paragraph: Laugaland et al. [2] cited for the progressive satisfaction trajectory.

Change in manuscript:

Discussion, all subsections: Multiple comparative and interpretive sentences inserted - see tracked changes in each subsection of the revised manuscript file.

Comment 6.16 - Discussion: Implications Without Citations

Please summarize the implications of the study without citations.

Response:

The Implications for Practice and Policy subsection has been revised. The citation '[19]' in the second implications paragraph has been removed. The self-efficacy mechanisms are now described in plain language as the study's applied conclusions, without attribution. All other implications paragraphs were already citation-free.

Change in manuscript:

Discussion, Implications subsection, paragraph 2: Revised to remove citation [19]. 'the self-efficacy mechanisms identified by Bandura [19]: mastery experiences...' -> 'the core sources of self-efficacy: mastery experiences...'

Comment 6.17 - Conclusion: Restructure to Describe Levels First

The conclusion should first briefly describe the levels of supervisory support, professional self-efficacy, and satisfaction before presenting the associations between these variables.

Response:

The opening paragraph of the Conclusion has been fully restructured. It now begins with observed levels of each construct (high supervisory support, high self-efficacy, high satisfaction; feedback and communication as most valued elements), then presents the associations and mediation finding, followed by the hospital-site variation, and concludes with the theoretical and contextual statements.

Change in manuscript:

Conclusion, paragraph 1: Replaced original opening with: 'Nursing students at KSU-affiliated teaching hospitals reported high levels of supervisory support, professional self-efficacy, and satisfaction with clinical placements. Among the three constructs, feedback quality emerged as the most valued supervisory element, while communication and team collaboration were the domains in which students expressed greatest professional confidence. Supervisory support was strongly and positively associated with both satisfaction and professional self-efficacy, and professional self-efficacy was independently associated with satisfaction, partially mediating the supervisory support-satisfaction relationship. Satisfaction varied significantly across hospital sites...'

Comment 6.18 - Language Quality

The manuscript contains several grammatical and linguistic errors. The authors are kindly requested to thoroughly revise the entire manuscript to improve academic language clarity, coherence, and scientific writing quality.

Response:

The manuscript has been carefully reviewed for grammatical and linguistic accuracy throughout. Key corrections include: (1) typographic error 'see Methods and and Appendix B' corrected to 'see Appendix B'; (2) comma splice in the Abstract Results sentence corrected; (3) spacing irregularities in the Discussion and Implications removed; (4) subject-verb agreement reviewed; (5) article usage reviewed throughout. All corrections are visible as tracked changes in the revised manuscript file.

Change in manuscript:

Throughout manuscript: Typographic error 'and and' corrected; extra spacing removed; minor grammatical corrections applied - all visible as tracked changes.

We believe the revised manuscript fully addresses all points raised by Reviewer #6 and meets the publication standards of PLOS ONE. We look forward to the Editor's decision.

Yours sincerely,

Dr. Abdulaziz Alodhailah
