# Supplementary material for: Exploring the relationship between supervisory support, self-efficacy, and satisfaction among nursing students in Saudi Arabia
Source: PLoS One. 2026 Jul 16;21(7):e0352318. doi: 10.1371/journal.pone.0352318 (PMC13375050; doi:10.1371/journal.pone.0352318)
Supplement: Peer review history

# Peer review: Decision Letter 0

**DOI:** 10.1371/journal.pone.0352318.r001

**Authors:** Majed Alamri

26 Feb 2026

PONE-D-26-01430

Exploring the Relationship Between Supervisory Support, Self-Efficacy, and Satisfaction Among Nursing Students in Saudi Arabia

PLOS One

Dear Dr. Alodhailah,

Thank you for submitting your manuscript to PLOS ONE. After careful consideration, we feel that it has merit but does not fully meet PLOS ONE’s publication criteria as it currently stands. Therefore, we invite you to submit a revised version of the manuscript that addresses the points raised during the review process.

Please address reviewers’ comments. Please submit your revised manuscript by Apr 12 2026 11:59PM. If you will need more time than this to complete your revisions, please reply to this message or contact the journal office at plosone@plos.org. When you're ready to submit your revision, log on to https://www.editorialmanager.com/pone/ and select the 'Submissions Needing Revision' folder to locate your manuscript file.

Please include the following items when submitting your revised manuscript:

- A letter that responds to each point raised by the academic editor and reviewer(s). You should upload this letter as a separate file labeled 'Response to Reviewers'.
- A marked-up copy of your manuscript that highlights changes made to the original version. You should upload this as a separate file labeled 'Revised Manuscript with Track Changes'.
- An unmarked version of your revised paper without tracked changes. You should upload this as a separate file labeled 'Manuscript'.

If you would like to make changes to your financial disclosure, please include your updated statement in your cover letter. Guidelines for resubmitting your figure files are available below the reviewer comments at the end of this letter.

If applicable, we recommend that you deposit your laboratory protocols in protocols.io to enhance the reproducibility of your results. Protocols.io assigns your protocol its own identifier (DOI) so that it can be cited independently in the future. For instructions see: https://journals.plos.org/plosone/s/submission-guidelines#loc-laboratory-protocols. Additionally, PLOS ONE offers an option for publishing peer-reviewed Lab Protocol articles, which describe protocols hosted on protocols.io. Read more information on sharing protocols at https://plos.org/protocols?utm_medium=editorial-email&utm_source=authorletters&utm_campaign=protocols.

We look forward to receiving your revised manuscript.

Kind regards,

Majed Sulaiman Alamri, PhD

Academic Editor

PLOS One

Journal Requirements:

When submitting your revision, we need you to address these additional requirements.

1. Please ensure that your manuscript meets PLOS ONE's style requirements, including those for file naming. The PLOS ONE style templates can be found at

https://journals.plos.org/plosone/s/file?id=wjVg/PLOSOne_formatting_sample_main_body.pdf and

https://journals.plos.org/plosone/s/file?id=ba62/PLOSOne_formatting_sample_title_authors_affiliations.pdf

2. Please update your submission to use the PLOS LaTeX template. The template and more information on our requirements for LaTeX submissions can be found at http://journals.plos.org/plosone/s/latex.

3. Thank you for stating the following in the Acknowledgments Section of your manuscript:

“This research did not receive any specific grant from funding agencies in the public, commercial, or not-for-profit sectors.”

We note that you have provided funding information that is not currently declared in your Funding Statement. However, funding information should not appear in the Acknowledgments section or other areas of your manuscript. We will only publish funding information present in the Funding Statement section of the online submission form.

Please remove any funding-related text from the manuscript and let us know how you would like to update your Funding Statement. Currently, your Funding Statement reads as follows:

“The author(s) received no specific funding for this work.”

Please include your amended statements within your cover letter; we will change the online submission form on your behalf.

4. We note that your Data Availability Statement is currently as follows: “All relevant data are within the manuscript and its Supporting Information files.”

Please confirm at this time whether or not your submission contains all raw data required to replicate the results of your study. Authors must share the “minimal data set” for their submission. PLOS defines the minimal data set to consist of the data required to replicate all study findings reported in the article, as well as related metadata and methods (https://journals.plos.org/plosone/s/data-availability#loc-minimal-data-set-definition).

For example, authors should submit the following data:

- The values behind the means, standard deviations and other measures reported;

- The values used to build graphs;

- The points extracted from images for analysis.

Authors do not need to submit their entire data set if only a portion of the data was used in the reported study.

If your submission does not contain these data, please either upload them as Supporting Information files or deposit them to a stable, public repository and provide us with the relevant URLs, DOIs, or accession numbers. For a list of recommended repositories, please see https://journals.plos.org/plosone/s/recommended-repositories.

If there are ethical or legal restrictions on sharing a de-identified data set, please explain them in detail (e.g., data contain potentially sensitive information, data are owned by a third-party organization, etc.) and who has imposed them (e.g., an ethics committee). Please also provide contact information for a data access committee, ethics committee, or other institutional body to which data requests may be sent. If data are owned by a third party, please indicate how others may request data access.

5. Your ethics statement should only appear in the Methods section of your manuscript. If your ethics statement is written in any section besides the Methods, please delete it from any other section.

6. If the reviewer comments include a recommendation to cite specific previously published works, please review and evaluate these publications to determine whether they are relevant and should be cited. There is no requirement to cite these works unless the editor has indicated otherwise.

[Note: HTML markup is below. Please do not edit.]

Reviewers' comments:

Reviewer's Responses to Questions

**Comments to the Author**

1. Is the manuscript technically sound, and do the data support the conclusions?

The manuscript must describe a technically sound piece of scientific research with data that supports the conclusions. Experiments must have been conducted rigorously, with appropriate controls, replication, and sample sizes. The conclusions must be drawn appropriately based on the data presented.

Reviewer #1: Partly

Reviewer #2: Yes

**********

2. Has the statistical analysis been performed appropriately and rigorously?

Reviewer #1: No

Reviewer #2: Yes

**********

3. Have the authors made all data underlying the findings in their manuscript fully available?

The PLOS Data policy requires authors to make all data underlying the findings described in their manuscript fully available without restriction, with rare exception (please refer to the Data Availability Statement in the manuscript PDF file). The data should be provided as part of the manuscript or its supporting information, or deposited to a public repository. For example, in addition to summary statistics, the data points behind means, medians and variance measures should be available. If there are restrictions on publicly sharing data—e.g. participant privacy or use of data from a third party—those must be specified.

Reviewer #1: Yes

Reviewer #2: Yes

**********

4. Is the manuscript presented in an intelligible fashion and written in standard English?

PLOS ONE does not copyedit accepted manuscripts, so the language in submitted articles must be clear, correct, and unambiguous. Any typographical or grammatical errors should be corrected at revision, so please note any specific errors here.

Reviewer #1: Yes

Reviewer #2: Yes

**********

5. Review Comments to the Author

Please use the space provided to explain your answers to the questions above. You may also include additional comments for the author, including concerns about dual publication, research ethics, or publication ethics. (Please upload your review as an attachment if it exceeds 20,000 characters)

Reviewer #1: The manuscript examines the relationship between supervisory support, professional self-efficacy, and satisfaction among undergraduate nursing students in Saudi Arabia. The topic is relevant, timely, and aligned with ongoing healthcare transformation efforts in the country. The study is clearly structured, theoretically grounded in social cognitive theory, and uses established instruments with good internal consistency.

However, several methodological and reporting issues require clarification before the manuscript is suitable for publication.

Abstract

- The abstract could be slightly shortened for conciseness while maintaining clarity.

Methods

- The Supervisory Support Scale and Professional Self-Efficacy Scale were adapted for the Saudi context. However:

- The process of cultural adaptation is not described in sufficient detail.

- It is unclear whether translation/back-translation was conducted.

- Construct validity (e.g., factor analysis) is not reported.

Analysis and Results

- The authors suggest partial mediation of professional self-efficacy in the relationship between supervisory support and satisfaction based on attenuation of regression coefficients. However:

- No formal mediation analysis (e.g., bootstrapped indirect effects using PROCESS or SEM) was conducted.

- The term “mediation” may be overstated given the cross-sectional design.

- Response rate is not reported. I would suggest report response rate.

- It is unclear how many students declined participation. I would suggest clarifying recruitment procedures and potential non-response bias.

- Assumptions for regression (normality, multicollinearity, homoscedasticity) are not reported.

- Effect sizes should be reported more consistently.

- Consider reporting confidence intervals alongside β coefficients in regression tables.

Discussion

- The Discussion section is somewhat lengthy and could be streamlined to reduce repetition, particularly regarding social cognitive theory.

References and editing

- There are some typographical issues:

- Repetition: “effectiveness effectiveness”

- Minor punctuation inconsistencies with semicolons

- Occasional spacing issues around citations. I would suggest checking all.

- Some references appear duplicated in citation numbering (e.g., [23] used multiple times in close proximity). Please verify reference accuracy and numbering.

Good luck!

Reviewer #2: Dear Authors,

Thank you for the opportunity to review this manuscript addressing a relevant and timely topic. The study demonstrates several notable strengths, including a clearly articulated research aim, an adequate sample size, the use of a stratified sampling approach, and transparent reporting of the regression analyses.

Although the stud is relevant and interesting, The authors used three scales, however, the scales were not translated into Arabic or no description of the translation process. Did you conduct a pilot study to assess if the tool fitting the Saudi Context. Were these scales used in another context?

Were you involved in teaching students? Please clarify who was responsible for data collection and what was the response rate?

You used the professional self-efficacy scale and mentioned that “Short form adapted from existing instruments”. However, original source unclear, item development process unclear, no content validation.

**********

6. PLOS authors have the option to publish the peer review history of their article (what does this mean?). If published, this will include your full peer review and any attached files.

If you choose “no”, your identity will remain anonymous but your review may still be made public.

**Do you want your identity to be public for this peer review?**  For information about this choice, including consent withdrawal, please see our Privacy Policy.

Reviewer #1: No

Reviewer #2: No

**********

[NOTE: If reviewer comments were submitted as an attachment file, they will be attached to this email and accessible via the submission site. Please log into your account, locate the manuscript record, and check for the action link "View Attachments". If this link does not appear, there are no attachment files.]

To ensure your figures meet our technical requirements, please review our figure guidelines: https://journals.plos.org/plosone/s/figures

You may also use PLOS’s free figure tool, NAAS, to help you prepare publication quality figures: https://journals.plos.org/plosone/s/figures#loc-tools-for-figure-preparation.

NAAS will assess whether your figures meet our technical requirements by comparing each figure against our figure specifications.

# Author comment: Author response to Decision Letter 1

**DOI:** 10.1371/journal.pone.0352318.r002

10 Mar 2026

Dear Editorial Team,

We are pleased to submit our substantially revised manuscript titled "Exploring the Relationship Between Supervisory Support, Self-Efficacy, and Satisfaction Among Nursing Students in Saudi Arabia" (PONE-D-26-01430) for consideration for publication in PLOS ONE.

We sincerely appreciate the constructive feedback provided by the academic editor and both reviewers. Their insightful comments have significantly strengthened our manuscript and our research. We have carefully and comprehensively addressed every point raised, and we detail our responses below.

MAJOR REVISIONS:

1. Formal Mediation Analysis (Critical Methodological Addition): In response to Reviewer #1's concern about the lack of formal mediation testing, we have conducted rigorous formal mediation analysis using PROCESS macro with 5,000 bootstrap resampling iterations. We now report:

• Direct, indirect, and total effects with 95% confidence intervals

• Formal statistical testing of the indirect effect

• Effect size (19% of total effect mediated)

• New Table 7 with complete mediation results

• Explicit discussion of cross-sectional design limitations

2. Instrument Adaptation and Validation Documentation: We have added comprehensive documentation of the translation and adaptation processes for all three scales, including:

• Forward-backward translation procedures

• Pilot study results (n=30)

• Confirmatory Factor Analysis validation (Appendix A)

• Content validity evidence for Professional Self-Efficacy Scale

3. Response Rate and Recruitment Transparency: We now report:

• 58% response rate (145/250 students)

• Detailed breakdown of declines and recruitment procedures

• Non-response bias assessment

4. Regression Assumption Testing: Comprehensive assumption testing added:

• Normality (Shapiro-Wilk tests): results provided

• Multicollinearity (VIF analysis): all values < 3.0

• Homoscedasticity (Breusch-Pagan test): confirmed

• Detailed results in Appendix B

5. Enhanced Statistical Reporting: All regression tables now include 95% confidence intervals and Cohen's f² effect sizes.

6. Discussion Streamlining: Discussion condensed by ~25% while maintaining substance; SCT discussion reduced from 4 to 2 mentions to eliminate repetition.

7. Typographical and Reference Corrections: All identified errors corrected; reference list audited.

8. Data Collection Team Clarification: Explicitly documented role separation to minimize bias.

9. Comprehensive Declarations Section: Revised to address all journal requirements including funding statement reconciliation and detailed data availability information.

INCLUDED SUBMISSION MATERIALS:

1. Response to Reviewers Letter - Detailed point-by-point responses to all reviewer and editorial comments

2. Revised Manuscript with Track Changes - All modifications clearly marked

3. Clean Manuscript - Final, polished version ready for publication

4. Appendices - Appendix A (CFA Results) and Appendix B (Assumption Testing)

SUMMARY OF CHANGES:

The revision addresses the most critical methodological concerns through formal mediation analysis while maintaining scientific rigor and transparency. The manuscript now clearly demonstrates:

• Rigorous statistical testing of mediation hypotheses

• Complete documentation of instrument validation

• Methodological transparency regarding assumptions and limitations

• Clear communication of findings within appropriate theoretical framework

We are confident that these substantial revisions meet PLOS ONE's publication standards and strengthen the contribution of our work to nursing education research.

Thank you for the opportunity to revise this work. We look forward to your response.

Sincerely,

Dr. Abdulaziz M. Alodhailah

Attachment Submitted filename: Response to Reviewers Letter.docx

# Peer review: Decision Letter 1

**DOI:** 10.1371/journal.pone.0352318.r003

**Authors:** Majed Alamri, Majed Alamri

16 Mar 2026

<div>PONE-D-26-01430R1

Exploring the Relationship Between Supervisory Support, Self-Efficacy, and Satisfaction Among Nursing Students in Saudi Arabia

PLOS One

Dear Dr. Alodhailah,

Thank you for submitting your manuscript to PLOS ONE. After careful consideration, we feel that it has merit but does not fully meet PLOS ONE’s publication criteria as it currently stands. Therefore, we invite you to submit a revised version of the manuscript that addresses the points raised during the review process.

Please address reviewer’s comments. Please submit your revised manuscript by Apr 30 2026 11:59PM. If you will need more time than this to complete your revisions, please reply to this message or contact the journal office at plosone@plos.org. When you're ready to submit your revision, log on to https://www.editorialmanager.com/pone/ and select the 'Submissions Needing Revision' folder to locate your manuscript file.

Please include the following items when submitting your revised manuscript:

- A letter that responds to each point raised by the academic editor and reviewer(s). You should upload this letter as a separate file labeled 'Response to Reviewers'.
- A marked-up copy of your manuscript that highlights changes made to the original version. You should upload this as a separate file labeled 'Revised Manuscript with Track Changes'.
- An unmarked version of your revised paper without tracked changes. You should upload this as a separate file labeled 'Manuscript'.

-->If you would like to make changes to your financial disclosure, please include your updated statement in your cover letter. Guidelines for resubmitting your figure files are available below the reviewer comments at the end of this letter.

If applicable, we recommend that you deposit your laboratory protocols in protocols.io to enhance the reproducibility of your results. Protocols.io assigns your protocol its own identifier (DOI) so that it can be cited independently in the future. For instructions see: https://journals.plos.org/plosone/s/submission-guidelines#loc-laboratory-protocols. Additionally, PLOS ONE offers an option for publishing peer-reviewed Lab Protocol articles, which describe protocols hosted on protocols.io. Read more information on sharing protocols at https://plos.org/protocols?utm_medium=editorial-email&utm_source=authorletters&utm_campaign=protocols.

We look forward to receiving your revised manuscript.

Kind regards,

Majed Sulaiman Alamri, PhD

Academic Editor

PLOS One

Journal Requirements:

If the reviewer comments include a recommendation to cite specific previously published works, please review and evaluate these publications to determine whether they are relevant and should be cited. There is no requirement to cite these works unless the editor has indicated otherwise.

Please review your reference list to ensure that it is complete and correct. If you have cited papers that have been retracted, please include the rationale for doing so in the manuscript text, or remove these references and replace them with relevant current references. Any changes to the reference list should be mentioned in the rebuttal letter that accompanies your revised manuscript. If you need to cite a retracted article, indicate the article’s retracted status in the References list and also include a citation and full reference for the retraction notice.

[Note: HTML markup is below. Please do not edit.]

Reviewers' comments:

Reviewer's Responses to Questions

**Comments to the Author**

1. If the authors have adequately addressed your comments raised in a previous round of review and you feel that this manuscript is now acceptable for publication, you may indicate that here to bypass the “Comments to the Author” section, enter your conflict of interest statement in the “Confidential to Editor” section, and submit your "Accept" recommendation.

Reviewer #1: (No Response)

Reviewer #2: All comments have been addressed

**********

2. Is the manuscript technically sound, and do the data support the conclusions?

The manuscript must describe a technically sound piece of scientific research with data that supports the conclusions. Experiments must have been conducted rigorously, with appropriate controls, replication, and sample sizes. The conclusions must be drawn appropriately based on the data presented.

Reviewer #1: Yes

Reviewer #2: Yes

**********

3. Has the statistical analysis been performed appropriately and rigorously?

Reviewer #1: Yes

Reviewer #2: Yes

**********

4. Have the authors made all data underlying the findings in their manuscript fully available?

The PLOS Data policy requires authors to make all data underlying the findings described in their manuscript fully available without restriction, with rare exception (please refer to the Data Availability Statement in the manuscript PDF file). The data should be provided as part of the manuscript or its supporting information, or deposited to a public repository. For example, in addition to summary statistics, the data points behind means, medians and variance measures should be available. If there are restrictions on publicly sharing data—e.g. participant privacy or use of data from a third party—those must be specified.

Reviewer #1: Yes

Reviewer #2: Yes

**********

5. Is the manuscript presented in an intelligible fashion and written in standard English?

PLOS ONE does not copyedit accepted manuscripts, so the language in submitted articles must be clear, correct, and unambiguous. Any typographical or grammatical errors should be corrected at revision, so please note any specific errors here.

Reviewer #1: Yes

Reviewer #2: Yes

**********

6. Review Comments to the Author

Please use the space provided to explain your answers to the questions above. You may also include additional comments for the author, including concerns about dual publication, research ethics, or publication ethics. (Please upload your review as an attachment if it exceeds 20,000 characters)

Reviewer #1: Thank you to the authors for the thorough revision. The manuscript has been substantially improved and most methodological concerns have been carefully addressed.

I have only some suggestions:

1. A minor typographical issue remains in the introduction (“effectiveness effectiveness”), which should be corrected.

2. The power analysis section refers to Cohen’s d (0.3). Since the study uses regression models, it may be more appropriate to report effect size in terms of f².

3. Given the cross-sectional design, I suggest slightly softening the interpretation of mediation to emphasize statistical mediation rather than causal inference.

Overall, the manuscript is now much stronger and makes a valuable contribution to understanding clinical supervision and student outcomes in nursing education.

Reviewer #2: Dear Authors,

Thank you for addressing all of my comments in the revised manuscript. I do not have any further comments and wish you all the best with the publication of your work.

**********

7. PLOS authors have the option to publish the peer review history of their article (what does this mean?). If published, this will include your full peer review and any attached files.

If you choose “no”, your identity will remain anonymous but your review may still be made public.

**Do you want your identity to be public for this peer review?**  For information about this choice, including consent withdrawal, please see our Privacy Policy.

Reviewer #1: No

Reviewer #2: No

**********

[NOTE: If reviewer comments were submitted as an attachment file, they will be attached to this email and accessible via the submission site. Please log into your account, locate the manuscript record, and check for the action link "View Attachments". If this link does not appear, there are no attachment files.]

To ensure your figures meet our technical requirements, please review our figure guidelines: https://journals.plos.org/plosone/s/figures

You may also use PLOS’s free figure tool, NAAS, to help you prepare publication quality figures: https://journals.plos.org/plosone/s/figures#loc-tools-for-figure-preparation.

NAAS will assess whether your figures meet our technical requirements by comparing each figure against our figure specifications.

# Author comment: Author response to Decision Letter 2

**DOI:** 10.1371/journal.pone.0352318.r004

17 Mar 2026

Response to Reviewers

Manuscript ID: PONE-D-26-01430R1

Title: Exploring the Relationship Between Supervisory Support, Self-Efficacy, and Satisfaction Among Nursing Students in Saudi Arabia

Authors: Thurayya Eid, Faihan F. Alshaibany, Abdulaziz M. Alodhailah, Waleed M. Alshehri

Dear Dr. Alamri and Reviewers,

We would like to sincerely thank you and the reviewers for the careful evaluation of our manuscript and for the constructive and encouraging feedback. We are pleased that the manuscript has been found to be substantially improved, technically sound, and suitable for publication pending minor revisions.

We have carefully addressed all remaining comments. All changes have been incorporated into the revised manuscript and are highlighted in the tracked changes version. Below, we provide a detailed, point-by-point response.

Reviewer #1

Comment 1: Typographical Error - Duplicated Word

A minor typographical issue remains in the introduction (“effectiveness effectiveness”), which should be corrected.

Response:

Thank you for identifying this typographical error. We have corrected the duplicated word in the Introduction section.

Comment 3: Statistical Reporting — Power Analysis Effect Size

The power analysis section refers to Cohen’s d (0.3). Since the study uses regression models, it may be more appropriate to report effect size in terms of f².

Response:

We have revised the power analysis section to report Cohen's f² instead of Cohen's d. Following the conversion formula $f^2 = \frac{d^2}{4}$ (Cohen, 1988, p. 276; Selya et al., 2012), the originally specified Cohen's d = 0.3 (medium effect) corresponds to:

$$f^2 = \frac{0.3^2}{4} = \frac{0.09}{4} = 0.0225$$

Change Made (Methods, Sample and Sampling section, page 6–7)

However, this would be a very small effect size for regression (below the f² = 0.02 "small" threshold is near, and the resulting sample would be very large). Given that we planned for a medium effect in regression, and our actual results confirmed medium-to-large effects (f² = 0.32 for supervisory support; overall model f² = 1.16), we have revised the power analysis to directly specify a medium effect size of f² = 0.15 (Cohen, 1988), which is standard for multiple regression. Using G*Power for linear multiple regression with f² = 0.15, α = 0.05, power = 0.80, and 7 predictors, the required minimum sample size is approximately 103. Our achieved sample of 145 exceeds this threshold and provides adequate statistical power.

Comment 3:

Given the cross-sectional design, I suggest slightly softening the interpretation of mediation to emphasize statistical mediation rather than causal inference.

Response:

Thank you for this insightful comment. We agree with this recommendation and have revised the relevant sections of the manuscript (Abstract, Results, and Discussion) to clarify that the mediation analysis reflects statistical associations rather than causal relationships. The language has been softened to avoid causal interpretations and to align with the limitations of a cross-sectional design.

Reviewer #2

Comment:

Thank you for addressing all of my comments in the revised manuscript. I do not have any further comments.

Response:

We sincerely thank the reviewer for their positive feedback and for their valuable input throughout the review process, which has helped strengthen the quality of our manuscript.

Additional Revisions

• We have carefully reviewed the entire manuscript to ensure clarity, consistency, and accuracy.

• Minor language edits have been made to improve readability.

We hope that the revised manuscript meets the journal’s requirements and is now suitable for publication in PLOS ONE. We appreciate your time and consideration.

Kind regards,

Dr. Abdulaziz M. Alodhailah

On behalf of all authors

King Saud University

Email: aalodhailah@ksu.edu.sa

Attachment Submitted filename: Response_to_Reviewers_Letter_auresp_2.docx

# Peer review: Decision Letter 2

**DOI:** 10.1371/journal.pone.0352318.r005

**Authors:** Majed Alamri, Majed Alamri, Majed Alamri

10 May 2026

PONE-D-26-01430R2

Exploring the Relationship Between Supervisory Support, Self-Efficacy, and Satisfaction Among Nursing Students in Saudi Arabia

PLOS One

Dear Dr. Alodhailah,

Thank you for submitting your manuscript to PLOS ONE. After careful consideration, we feel that it has merit but does not fully meet PLOS ONE’s publication criteria as it currently stands. Therefore, we invite you to submit a revised version of the manuscript that addresses the points raised during the review process.

Please address reviewer’s comments.

Please submit your revised manuscript by Jun 24 2026 11:59PM. If you will need more time than this to complete your revisions, please reply to this message or contact the journal office at plosone@plos.org. When you're ready to submit your revision, log on to https://www.editorialmanager.com/pone/ and select the 'Submissions Needing Revision' folder to locate your manuscript file.

Please include the following items when submitting your revised manuscript:

- A letter that responds to each point raised by the academic editor and reviewer(s). You should upload this letter as a separate file labeled 'Response to Reviewers'.
- A marked-up copy of your manuscript that highlights changes made to the original version. You should upload this as a separate file labeled 'Revised Manuscript with Track Changes'.
- An unmarked version of your revised paper without tracked changes. You should upload this as a separate file labeled 'Manuscript'.

If you would like to make changes to your financial disclosure, please include your updated statement in your cover letter. Guidelines for resubmitting your figure files are available below the reviewer comments at the end of this letter.

If applicable, we recommend that you deposit your laboratory protocols in protocols.io to enhance the reproducibility of your results. Protocols.io assigns your protocol its own identifier (DOI) so that it can be cited independently in the future. For instructions see: https://journals.plos.org/plosone/s/submission-guidelines#loc-laboratory-protocols. Additionally, PLOS ONE offers an option for publishing peer-reviewed Lab Protocol articles, which describe protocols hosted on protocols.io. Read more information on sharing protocols at https://plos.org/protocols?utm_medium=editorial-email&utm_source=authorletters&utm_campaign=protocols.

As the corresponding author, your ORCID iD is verified in the submission system and will appear in the published article. PLOS supports the use of ORCID, and we encourage all coauthors to register for an ORCID iD and use it as well. Please encourage your coauthors to verify their ORCID iD within the submission system before final acceptance, as unverified ORCID iDs will not appear in the published article. *Only*  the individual author can complete the verification step; PLOS staff *cannot*  verify ORCID iDs on behalf of authors.

We look forward to receiving your revised manuscript.

Kind regards,

Majed Sulaiman Alamri, PhD

Academic Editor

PLOS One

Journal Requirements:

If the reviewer comments include a recommendation to cite specific previously published works, please review and evaluate these publications to determine whether they are relevant and should be cited. There is no requirement to cite these works unless the editor has indicated otherwise.

[Note: HTML markup is below. Please do not edit.]

Reviewers' comments:

Reviewer's Responses to Questions

**Comments to the Author**

1. If the authors have adequately addressed your comments raised in a previous round of review and you feel that this manuscript is now acceptable for publication, you may indicate that here to bypass the “Comments to the Author” section, enter your conflict of interest statement in the “Confidential to Editor” section, and submit your "Accept" recommendation.

Reviewer #1: All comments have been addressed

Reviewer #2: All comments have been addressed

Reviewer #3: All comments have been addressed

Reviewer #4: All comments have been addressed

Reviewer #5: All comments have been addressed

Reviewer #6: All comments have been addressed

**********

2. Is the manuscript technically sound, and do the data support the conclusions?

The manuscript must describe a technically sound piece of scientific research with data that supports the conclusions. Experiments must have been conducted rigorously, with appropriate controls, replication, and sample sizes. The conclusions must be drawn appropriately based on the data presented.

Reviewer #1: Yes

Reviewer #2: Yes

Reviewer #3: Yes

Reviewer #4: Yes

Reviewer #5: Yes

Reviewer #6: Partly

**********

3. Has the statistical analysis been performed appropriately and rigorously?

Reviewer #1: Yes

Reviewer #2: Yes

Reviewer #3: Yes

Reviewer #4: I Don't Know

Reviewer #5: Yes

Reviewer #6: No

**********

4. Have the authors made all data underlying the findings in their manuscript fully available?

The PLOS Data policy requires authors to make all data underlying the findings described in their manuscript fully available without restriction, with rare exception (please refer to the Data Availability Statement in the manuscript PDF file). The data should be provided as part of the manuscript or its supporting information, or deposited to a public repository. For example, in addition to summary statistics, the data points behind means, medians and variance measures should be available. If there are restrictions on publicly sharing data—e.g. participant privacy or use of data from a third party—those must be specified.

Reviewer #1: (No Response)

Reviewer #2: Yes

Reviewer #3: Yes

Reviewer #4: Yes

Reviewer #5: Yes

Reviewer #6: Yes

**********

5. Is the manuscript presented in an intelligible fashion and written in standard English?

PLOS ONE does not copyedit accepted manuscripts, so the language in submitted articles must be clear, correct, and unambiguous. Any typographical or grammatical errors should be corrected at revision, so please note any specific errors here.

Reviewer #1: Yes

Reviewer #2: Yes

Reviewer #3: Yes

Reviewer #4: Yes

Reviewer #5: Yes

Reviewer #6: No

**********

6. Review Comments to the Author

Please use the space provided to explain your answers to the questions above. You may also include additional comments for the author, including concerns about dual publication, research ethics, or publication ethics. (Please upload your review as an attachment if it exceeds 20,000 characters)

Reviewer #1: Thank you for addressing the comments. The authors have addressed the remaining comments carefully and thoughtfully. Good Luck!

Reviewer #2: Dear Authors,

Thank you for revising the manuscript and enhancing its overall quality. I have no further comments at this stage and wish you all the best with your publication.

Reviewer #3: I congratulate the authors on their meticulous and detailed work.

I suggest that the authors specify the research design as "cross-sectional correlational".

Reviewer #4: The authors have adequately addressed all of the concerns raised in the previous round of review. The revised manuscript shows clear and appropriate revisions, with responses that are thorough and well‑justified. The clarifications provided in the methods and analyses have improved the overall rigour and transparency of the study, and the revisions have strengthened the alignment between the results and conclusions. Overall, I am satisfied that reviewers' comments have been fully addressed, and I believe the manuscript is now suitable for publication.

Reviewer #5: The manuscript has undergone two previous rounds of revision, and the authors have adequately addressed the comments raised by the reviewers. In its current form, the presentation of the article is suitable for publication.

Furthermore, the context of nursing education in the Gulf region presents unique characteristics. Studies like this provide valuable insights to the research community by highlighting the diverse approaches adopted in nursing higher education within this region.

Reviewer #6: (No Response)

**********

7. PLOS authors have the option to publish the peer review history of their article (what does this mean?). If published, this will include your full peer review and any attached files.

If you choose “no”, your identity will remain anonymous but your review may still be made public.

**Do you want your identity to be public for this peer review?**  For information about this choice, including consent withdrawal, please see our Privacy Policy.

Reviewer #1: No

Reviewer #2: No

Reviewer #3: No

Reviewer #4: **Yes:** Dr Adeniyi Olanrewaju Adeleye

Reviewer #5: No

Reviewer #6: No

**********

[NOTE: If reviewer comments were submitted as an attachment file, they will be attached to this email and accessible via the submission site. Please log into your account, locate the manuscript record, and check for the action link "View Attachments". If this link does not appear, there are no attachment files.]

To ensure your figures meet our technical requirements, please review our figure guidelines: https://journals.plos.org/plosone/s/figures

You may also use PLOS’s free figure tool, NAAS, to help you prepare publication quality figures: https://journals.plos.org/plosone/s/figures#loc-tools-for-figure-preparation.

NAAS will assess whether your figures meet our technical requirements by comparing each figure against our figure specifications.

Attachment Submitted filename: Upload Reviewer Attachment.pdf

# Author comment: Author response to Decision Letter 3

**DOI:** 10.1371/journal.pone.0352318.r006

14 May 2026

POINT-BY-POINT RESPONSE TO REVIEWER COMMENTS

Manuscript Title: Supervisory Support, Professional Self-Efficacy, and Student Satisfaction in Nursing Clinical Education: A Cross-Sectional Study in Saudi Arabia

The authors thank the reviewer for their thorough and constructive feedback. We have addressed each comment systematically below. All revisions are reflected in the tracked-changes manuscript. We believe the manuscript has been substantially strengthened as a result.

ABSTRACT

# Reviewer Comment Authors’ Response

A.1 Please provide a more concise summary of the study background. Done. The abstract background has been condensed to four sentences that convey the core rationale without restating the introduction in detail.

A.2 The general objective of the study should be explicitly stated at the end of the background section. The revised abstract background now concludes with an explicit statement of the study’s aim.

A.3 Clearly state the study design in complete form, e.g., ‘This descriptive cross-sectional study was conducted among 145 nursing students at King Saud University hospitals.’ The Methods sentence now reads: “This descriptive cross-sectional study was conducted among 145 nursing students (second-, third-, and fourth-year bachelor’s degree students) at three hospitals affiliated with King Saud University in Riyadh, Saudi Arabia.”

A.4 ‘Descriptive statistics summarized study variables’ is too general. Specify the key study variables. Revised to: “Descriptive statistics summarised the three key study variables (supervisory support, professional self-efficacy, and student satisfaction) alongside sociodemographic characteristics.”

A.5 ‘Pearson correlations examined relationships’ should be revised to reflect the purpose more accurately. Revised to: “Pearson correlation coefficients assessed the strength and direction of linear associations among continuous study variables.”

A.6 Key socio-demographic characteristics should be briefly presented before reporting main results. Added to abstract Results: “Of 250 eligible students, 145 completed the questionnaire (response rate = 58%). The sample was predominantly female (60.7%) and aged 20–22 years (66.9%).”

A.7 Provide the response rate at the beginning of the Results section. Response rate (58%, 145/250) is now the opening statement of the abstract Results section.

A.8 Clarify what reported mean scores are based on (e.g., ‘out of 5’) and ensure consistent reporting of means with SD. All means in the abstract are now reported as “mean = X.XX ± SD out of 5.”

A.9 Key regression results should be clearly and concisely stated. The abstract now states: “In regression analysis, supervisory support remained significantly associated with satisfaction (β = 0.43, p < 0.001) even after accounting for professional self-efficacy, consistent with partial statistical mediation.”

BACKGROUND

# Reviewer Comment Authors’ Response

B.1 Include a dedicated paragraph discussing key challenges and barriers related to supervisory support and professional self-efficacy in nursing education. A new paragraph has been added to the Background detailing challenges including high instructor workloads, variability in supervision quality, limited feedback opportunities, and student–supervisor communication gaps, with supporting citations [3, 5, 21].

B.2 ‘The quality of supervisory support is paramount’ should be strengthened with concrete examples of how supervisory support influences outcomes. The relevant sentence now cites specific outcomes: clinical skill development, professional identity formation, and transition to practice readiness.

B.3 Several paragraphs contain grammatical and stylistic issues. The entire Background section has been revised for grammatical accuracy, formal academic register, and stylistic consistency throughout.

B.4 The role of clinical instructors should be elaborated to highlight direct influence on learning outcomes and clinical competence. Revised to: “…clinical instructors… play a direct role in shaping clinical competence, professional identity, and readiness for independent practice [9].”

B.5 The hypothesis statement should be clearly positioned immediately before the objectives. The hypothesis sentence (“Guided by social cognitive theory, we hypothesised…”) now immediately precedes the study objectives at the end of the Background.

B.6 Clearly explain how Social Cognitive Theory guided variable selection and how constructs were operationalised. A new sentence now explains that supervisory feedback operationalises verbal persuasion, practitioner observation operationalises vicarious learning, and graduated practice operationalises mastery experience, per Bandura [19].

B.7 A dedicated paragraph should be added discussing the potential impact of the study on clinical practice, education, management, and research. Added. The new paragraph addresses: clinical practice (supervision quality and patient care), education (curriculum and student support), management (supervisor training and placement organisation), and research (future interventional and longitudinal studies).

METHODS

# Reviewer Comment Authors’ Response

M.1 Summarize the study design section more concisely while maintaining essential details; align with STROBE. Revised to one concise paragraph: “This descriptive cross-sectional study examined associations among supervisory support, professional self-efficacy, and nursing student satisfaction. Reporting adheres to STROBE guidelines.”

M.2 Specify the number and names of the hospitals affiliated with King Saud University and justify their selection. The Setting section now names all three hospitals (KSUMC, KKUH, KAUH) and provides explicit justification (accessibility, structured training programmes, representativeness of the KSU system).

M.3 The rationale for a single-university setting should be stated; this is a limitation. The rationale is noted in Methods (representativeness of KSU clinical system) and the limitation of single-university generalisability is explicitly acknowledged in the Strengths and Limitations section.

M.4 Specify the assumed attrition rate and avoid repetition of the non-response explanation. Revised to specify an estimated 10–15% non-response rate as the basis for increasing the target to 145. The duplicated sentence has been removed.

M.5 Report the total target population size with a reference; describe the formula for sample size estimation. Population size (approximately 250 students enrolled during the study period) is reported. G*Power 3.1 and the parameters for a priori power analysis are now explicitly described with citation [44].

M.6 Clarify the stratified random sampling procedure, including strata definition and allocation. The Methods now describes stratification by year of study (second, third, fourth year) and states that proportionate allocation was used to ensure representation at each stage.

M.7 ‘Students who had completed at least one clinical rotation’ — clarify terminology. Revised to: “having completed at least one supervised clinical placement in a hospital department under the university curriculum.”

M.8 Inclusion criteria should include academic level and educational level. Inclusion criteria now explicitly state: “enrolled in the second, third, or fourth year of the KSU bachelor’s degree nursing programme.”

M.9 Specify the exact nature of the data collection tools. The Methods now states that instruments were structured self-administered questionnaires administered in paper form.

M.10 The tool selection rationale is too general; explain psychometric properties more clearly. The Data Collection Instruments section now explains how Cronbach’s α, CFA fit indices, and prior validation evidence informed instrument selection.

M.11 The sentence explaining subgroup analysis context is not essential and should be removed. Removed.

M.12 Provide a detailed description of the translation/adaptation process, number and expertise of experts, and modifications made. Also clarify why construct validity was assessed after data collection. The adaptation process is now described in detail: forward translation by two bilingual nursing education experts, backward translation by two independent translators, consensus review, pilot testing with n = 30 students. The justification for post-collection CFA (full sample required for adequate CFA power) is noted.

M.13 Explain how levels (high/moderate/low) were determined. Now specified: trisection of the 1–5 Likert scale, with low ≤ 2.33, moderate 2.34–3.66, high ≥ 3.67, consistent with published scoring conventions [24].

M.14 Data collection procedures are described more than once; merge under a single subheading. All data collection content consolidated under a single “Data Collection Procedure” subheading.

M.15 Non-response bias statement requires clarification. Clarified: χ² comparisons of age group, gender, and year of study between respondents and non-respondents using institutional enrolment records are now reported with test statistics and p-values.

M.16 Recalculate and clearly report response rate based on completed questionnaires / sampled participants. Response rate = 145/250 = 58% is now the first sentence of the non-response bias paragraph.

M.17 Data collection sentence should use formal academic language. Revised: “Questionnaires were administered face-to-face at the three participating hospitals by trained research assistants.”

M.18 Statistical analysis section should be summarised into one coherent paragraph. Consolidated into a single paragraph covering all statistical methods, significance thresholds, and software.

M.19 Correlation analysis statement is not accurate — Pearson should be limited to continuous variables. Revised. The Methods now states that Pearson correlation assessed linear associations among continuous variables only, and notes that the binary gender variable’s correlation with continuous outcomes should be interpreted cautiously.

RESULTS

# Reviewer Comment Authors’ Response

R.1 Report whether assumptions for Pearson correlation, regression, and ANOVA were assessed before analysis. Added to Results opening: “All regression model assumptions were satisfied prior to analysis (see Methods and Appendix B).” The Methods section now contains a full Regression Model Assumptions Testing subsection.

R.2 Clarify rationale for including age and gender in correlation analysis. Age (continuous) is appropriate for Pearson correlation. Gender (binary) is now flagged in both the correlation table footnote and text as a descriptive-only measure interpreted with caution.

R.3 Write the name of the table at the end of each relevant paragraph. Each results paragraph now ends with a parenthetical table citation, e.g., (Table 6).

R.4 Table numbering is duplicated (Table 9 appears twice). Corrected. The ANOVA table is now Table 10; all table numbers are sequential and consistent throughout.

R.5 Provide a concise narrative summary for each table. Each table is accompanied by 1–2 narrative paragraphs highlighting key findings. Summary language is in the text; tables present the full data.

R.6 Ensure interpretation of correlation coefficients follows accepted guidelines. Correlation magnitudes are now described using accepted benchmarks (r ≈ 0.50 = moderate, r ≥ 0.60 = strong) consistent with standard conventions.

R.7 Some statements contain interpretation rather than pure reporting (e.g., ‘likely due to increased familiarity’). Move to Discussion. Interpretive language has been removed from Results and incorporated into the Discussion.

R.8 Mediation findings should be interpreted as statistical mediation, not causal. Throughout the Results and Discussion, all mediation language is qualified: “statistical mediation,” “statistical association,” and the cross-sectional design limitation is noted explicitly in the table footnote.

R.9 Explain how categorical variables were coded for regression models. Now specified: “Gender, year of study, and clinical placement location were dummy-coded for entry into regression models.” This appears in the Methods and is noted in Table 6 footnotes.

R.10 Since ANOVA showed significant differences, provide post-hoc comparisons (e.g., Tukey). Post-hoc Tukey test results are now reported for both year-of-study and clinical placement location comparisons (Table 9 footnotes and Results narrative).

R.11 Verify consistency between text, tables, beta coefficients, p-values, and CIs. All values have been cross-checked for consistency between narrative text and tables. No discrepancies remain.

R.12 Clarify whether standardised or unstandardised coefficients are reported. Clarified throughout: β = standardised regression coefficients in all tables and text. This is noted in all table footnotes.

R.13 Findings are repeated multiple times in text and tables. Reduce redundancy. Redundant restatements have been removed. The text summarises key findings; tables present full data.

R.14 State whether mediation assumptions were assessed and whether PROCESS included covariates. Added to Results: mediation assumptions are described (paths a, b significant; total effect significant). PROCESS Model 4 is noted; no additional covariates beyond the mediator were included in the PROCESS model.

R.15 Report effect sizes for all major analyses. Cohen’s f² is reported for all regression models (Table 6). η² is reported for the ANOVA (Table 10 footnote). Correlation coefficients serve as their own effect size metrics.

R.16 Describe missing data and how managed. Added: “Missing data were negligible: no item-level missing data were observed, as research assistants verified questionnaire completeness at the point of collection.”

R.17 Some tables are overly dense; improve formatting and alignment of decimal places. Tables have been reformatted with consistent decimal alignment, adequate cell padding, and simplified structure where possible.

R.18 Causal language (influencing, effect, predictors) should be replaced with associative language. All instances of ‘influence,’ ‘effect,’ and ‘predict’ in the Results have been replaced with ‘associated with,’ ‘explained variance,’ or equivalent associative terminology.

DISCUSSION

# Reviewer Comment Authors’ Response

D.1 Discussion should focus on interpretation, not restatement of statistical values. The Discussion has been restructured to emphasise theoretical interpretation and contextualisation. Specific statistical values are cited only where necessary for interpretive precision; detailed results remain in the Results section.

D.2 Causal language (influence, predictor, fosters) should be replaced with associative terminology. All causal language in the Discussion has been replaced with ‘associated with,’ ‘statistically mediated,’ and similar appropriate phrasing.

D.3 Several findings are discussed without sufficient comparison to regional or international studies. Each thematic subsection now includes direct comparison with international evidence (Singapore [13], US [14]) and acknowledges where findings align with or diverge from prior research.

D.4 Social Cognitive Theory is mentioned but the discussion should critically explain how findings support or extend it. A new paragraph explicitly explains how study findings instantiate SCT mechanisms: mastery experience, vicarious learning, and verbal persuasion, and how partial mediation extends the theory empirically.

D.5 Discussion repeatedly restates statistical values from Results. Removed. The Discussion now references Results figures minimally and only where they anchor an interpretive point.

D.6 Cultural, educational, and institutional characteristics in Saudi Arabia should be discussed more clearly. A dedicated paragraph now addresses hierarchical organisational culture, gender dynamics in clinical settings, and institutional change under Vision 2030 as contextual factors shaping the findings.

D.7 Inter-hospital differences are mentioned briefly; provide more in-depth discussion of contributing factors. The inter-hospital variation is now discussed in a dedicated subsection covering supervisory practices, resource availability, staffing ratios, and organisational culture as plausible explanatory factors.

D.8 Acknowledge potential biases (self-repor

Attachment Submitted filename: Response_to_Reviewers.docx

# Peer review: Decision Letter 3

**DOI:** 10.1371/journal.pone.0352318.r007

**Authors:** Majed Alamri, Majed Alamri, Majed Alamri, Majed Alamri

25 May 2026

<div>PONE-D-26-01430R3

Exploring the Relationship Between Supervisory Support, Self-Efficacy, and Satisfaction Among Nursing Students in Saudi Arabia

PLOS One

Dear Dr. Alodhailah,

Thank you for submitting your manuscript to PLOS ONE. After careful consideration, we feel that it has merit but does not fully meet PLOS ONE’s publication criteria as it currently stands. Therefore, we invite you to submit a revised version of the manuscript that addresses the points raised during the review process.

Please submit your revised manuscript by Jul 09 2026 11:59PM. If you will need more time than this to complete your revisions, please reply to this message or contact the journal office at plosone@plos.org. When you're ready to submit your revision, log on to https://www.editorialmanager.com/pone/ and select the 'Submissions Needing Revision' folder to locate your manuscript file.

Please include the following items when submitting your revised manuscript:

- A letter that responds to each point raised by the academic editor and reviewer(s). You should upload this letter as a separate file labeled 'Response to Reviewers'.
- A marked-up copy of your manuscript that highlights changes made to the original version. You should upload this as a separate file labeled 'Revised Manuscript with Track Changes'.
- An unmarked version of your revised paper without tracked changes. You should upload this as a separate file labeled 'Manuscript'.

If you would like to make changes to your financial disclosure, please include your updated statement in your cover letter. Guidelines for resubmitting your figure files are available below the reviewer comments at the end of this letter.

If applicable, we recommend that you deposit your laboratory protocols in protocols.io to enhance the reproducibility of your results. Protocols.io assigns your protocol its own identifier (DOI) so that it can be cited independently in the future. For instructions see: https://journals.plos.org/plosone/s/submission-guidelines#loc-laboratory-protocols. Additionally, PLOS ONE offers an option for publishing peer-reviewed Lab Protocol articles, which describe protocols hosted on protocols.io. Read more information on sharing protocols at https://plos.org/protocols?utm_medium=editorial-email&utm_source=authorletters&utm_campaign=protocols.

As the corresponding author, your ORCID iD is verified in the submission system and will appear in the published article. PLOS supports the use of ORCID, and we encourage all coauthors to register for an ORCID iD and use it as well. Please encourage your coauthors to verify their ORCID iD within the submission system before final acceptance, as unverified ORCID iDs will not appear in the published article. *Only*  the individual author can complete the verification step; PLOS staff *cannot*  verify ORCID iDs on behalf of authors.

We look forward to receiving your revised manuscript.

Kind regards,

Majed Sulaiman Alamri, PhD

Academic Editor

PLOS One

Journal Requirements:

If the reviewer comments include a recommendation to cite specific previously published works, please review and evaluate these publications to determine whether they are relevant and should be cited. There is no requirement to cite these works unless the editor has indicated otherwise.

Please review your reference list to ensure that it is complete and correct. If you have cited papers that have been retracted, please include the rationale for doing so in the manuscript text, or remove these references and replace them with relevant current references. Any changes to the reference list should be mentioned in the rebuttal letter that accompanies your revised manuscript. If you need to cite a retracted article, indicate the article’s retracted status in the References list and also include a citation and full reference for the retraction notice.

[Note: HTML markup is below. Please do not edit.]

Reviewers' comments:

Reviewer's Responses to Questions

**Comments to the Author**

1. If the authors have adequately addressed your comments raised in a previous round of review and you feel that this manuscript is now acceptable for publication, you may indicate that here to bypass the “Comments to the Author” section, enter your conflict of interest statement in the “Confidential to Editor” section, and submit your "Accept" recommendation.

Reviewer #1: All comments have been addressed

Reviewer #4: All comments have been addressed

Reviewer #6: (No Response)

**********

2. Is the manuscript technically sound, and do the data support the conclusions?

The manuscript must describe a technically sound piece of scientific research with data that supports the conclusions. Experiments must have been conducted rigorously, with appropriate controls, replication, and sample sizes. The conclusions must be drawn appropriately based on the data presented.

Reviewer #1: Yes

Reviewer #4: Yes

Reviewer #6: Partly

**********

3. Has the statistical analysis been performed appropriately and rigorously?

Reviewer #1: I Don't Know

Reviewer #4: I Don't Know

Reviewer #6: No

**********

4. Have the authors made all data underlying the findings in their manuscript fully available?

The PLOS Data policy requires authors to make all data underlying the findings described in their manuscript fully available without restriction, with rare exception (please refer to the Data Availability Statement in the manuscript PDF file). The data should be provided as part of the manuscript or its supporting information, or deposited to a public repository. For example, in addition to summary statistics, the data points behind means, medians and variance measures should be available. If there are restrictions on publicly sharing data—e.g. participant privacy or use of data from a third party—those must be specified.

Reviewer #1: Yes

Reviewer #4: Yes

Reviewer #6: Yes

**********

5. Is the manuscript presented in an intelligible fashion and written in standard English?

PLOS ONE does not copyedit accepted manuscripts, so the language in submitted articles must be clear, correct, and unambiguous. Any typographical or grammatical errors should be corrected at revision, so please note any specific errors here.

Reviewer #1: Yes

Reviewer #4: Yes

Reviewer #6: No

**********

6. Review Comments to the Author

Please use the space provided to explain your answers to the questions above. You may also include additional comments for the author, including concerns about dual publication, research ethics, or publication ethics. (Please upload your review as an attachment if it exceeds 20,000 characters)

Reviewer #1: Thank you for the revised manuscript. The authors have addressed the majority of the previous comments thoroughly, and the manuscript has improved substantially in methodological clarity, theoretical integration, statistical reporting, and overall presentation.

Reviewer #4: Thank you for providing further revision on this paper. Note that I have not reviewed any statistical analysis associated with your study.

Reviewer #6: The authors are commended for their efforts in revising the manuscript and addressing the previous comments. However, several issues remain that require further major revision to improve clarity, methodological rigor, statistical reporting, and academic writing quality. The following comments are provided to further strengthen the manuscript. Most importantly, the Discussion section requires substantial revision and a comprehensive rewrite, with a stronger emphasis on detailed comparison with relevant previous studies.

The manuscript contains several grammatical and linguistic errors. The authors are kindly requested to thoroughly revise the entire manuscript to improve academic language clarity, coherence, and scientific writing quality.

Abstract

It is not necessary to include the names of the hospitals in the abstract. Please remove them from the abstract.

The response rate calculation requires revision. It should be clearly based on the appropriate sampling frame rather than the total population. Please recheck the calculation and ensure consistency between the sample size determination and the reported response rate.

Background

Please add a supporting citation for the following statement: "Effective supervision creates environments where students develop critical thinking, clinical decision-making, and hands-on nursing skills.”

Provide appropriate citations for the following statements: "Despite broad consensus on the value of supervision, significant challenges persist. Clinical instructors often face high workloads and variable caseloads that limit individualized student contact. Supervision quality varies considerably across sites owing to differences in instructor experience, institutional resources, and organizational culture.”

The authors need to clarify the following statement by specifying the mechanisms and adding appropriate citations: "In the clinical education context, these mechanisms are operationalized through supervisor feedback (verbal persuasion), direct observation of skilled practitioners (vicarious learning), and graduated practice under supportive conditions (mastery experience).”

Methods

The sample size is reported as 103 based on calculation and later increased to 145 participants. However, the reported response rate of 58% requires clarification and revision. Please ensure consistency between the sample size calculation, population size, and final included participants.

The total population size (250 students) must be properly referenced.

The authors have calculated the sample size based on calculation, so I suggest you remove the attached paragraph from the methods section "Response rate and non-response bias. Of approximately 250 students enrolled in clinical placements at the three hospitals during the study period, 145 returned valid questionnaires (response rate = 58%). To assess potential non-response bias, available demographic characteristics (age group, gender, year of study) of respondents and non-respondents were compared using institutional enrolment records. No statistically significant differences were found (χ² age = 1.23, p = 0.267; χ² gender = 0.91, p = 0.341; χ² year = 1.45, p = 0.485), suggesting the final sample was reasonably representative of the broader population".

The following statement should be revised for clarity and accuracy: "Of approximately 250 students enrolled in clinical placements at the three hospitals during the study period, 145 returned valid questionnaires (response rate = 58%).”

Results

Please move the provided paragraph related to regression analysis to the appropriate section and remove “ See Methods and and Appendix B.”

The authors should write the accurate response rate in the first sentence in the results section.

The authors must write the number of several tables at the end of the paragraphs, not in the first sentence, such as "Table 1 presents the sociodemographic characteristics of the sample (n = 145).

In Table 9, ANOVA p-values are reported for variables where ANOVA is not appropriate for several variables such as gender. Please reanalyze the data using appropriate statistical tests.

Discussion

This section requires substantial revision, as its current quality is insufficient for publication standards. Note: The discussion should be rewritten in a more coherent, critical, and evidence-based academic style.

Avoid overemphasis on numerical values within the discussion section such as "The significant variation in satisfaction across the three hospitals (ANOVA: F = 4.312, p = 0.016; η² = 0.058) warrants particular attention".

Several subsections lack adequate comparison with existing literature. Please strengthen the discussion by:

Comparing findings with relevant previous studies;

Providing scientific justification for key results;

Strengthening theoretical and empirical interpretation.

Please summarize the implications of the study without citations.

Conclusion

The conclusion should first briefly describe the levels of supervisory support, professional self-efficacy, and satisfaction before presenting the associations between these variables.

**********

7. PLOS authors have the option to publish the peer review history of their article (what does this mean?). If published, this will include your full peer review and any attached files.

If you choose “no”, your identity will remain anonymous but your review may still be made public.

**Do you want your identity to be public for this peer review?**  For information about this choice, including consent withdrawal, please see our Privacy Policy.

Reviewer #1: No

Reviewer #4: No

Reviewer #6: No

**********

[NOTE: If reviewer comments were submitted as an attachment file, they will be attached to this email and accessible via the submission site. Please log into your account, locate the manuscript record, and check for the action link "View Attachments". If this link does not appear, there are no attachment files.]

To ensure your figures meet our technical requirements, please review our figure guidelines: https://journals.plos.org/plosone/s/figures

You may also use PLOS’s free figure tool, NAAS, to help you prepare publication quality figures: https://journals.plos.org/plosone/s/figures#loc-tools-for-figure-preparation.

NAAS will assess whether your figures meet our technical requirements by comparing each figure against our figure specifications.

# Author comment: Author response to Decision Letter 4

**DOI:** 10.1371/journal.pone.0352318.r008

25 May 2026

Response to Reviewers

Manuscript ID: PONE-D-26-01430R3

Dear Dr. Majed Alamri and Members of the Editorial Board,

We sincerely thank the Editor and all three Reviewers for their careful reading of our revised manuscript entitled "Exploring the Relationship Between Supervisory Support, Self-Efficacy, and Satisfaction Among Nursing Students in Saudi Arabia." We have thoroughly revised the manuscript in response to every point raised. Below we provide a point-by-point account of all changes made. All revisions are indicated as tracked changes attributed to Dr. Abdulaziz Alodhailah in the accompanying Revised Manuscript with Track Changes file.

Reviewer #1

Comment 1.1

Thank you for the revised manuscript. The authors have addressed the majority of the previous comments thoroughly, and the manuscript has improved substantially in methodological clarity, theoretical integration, statistical reporting, and overall presentation. All comments have been addressed.

Response:

We thank Reviewer #1 for this positive assessment. The current revision further addresses all remaining points raised by Reviewer #6, as detailed below.

Reviewer #4

Comment 4.1

Thank you for providing further revision on this paper. Note that I have not reviewed any statistical analysis associated with your study. All comments have been addressed.

Response:

We thank Reviewer #4. Statistical concerns raised in this round by Reviewer #6 have been fully addressed, including re-analysis of group comparisons in Table 9 using appropriate tests (see Comment 6.10 below).

Reviewer #6

General Comment

The authors are commended for their efforts. However, several issues remain that require further major revision to improve clarity, methodological rigour, statistical reporting, and academic writing quality. The Discussion section requires substantial revision and a comprehensive rewrite, with a stronger emphasis on detailed comparison with relevant previous studies. The manuscript contains several grammatical and linguistic errors.

Response:

We thank Reviewer #6 for this thorough critique. We have systematically revised every section of the manuscript in response. The Discussion has been substantially expanded with direct comparisons to prior studies, and grammatical clarity has been improved throughout. Each specific point is addressed below.

Comment 6.1 - Abstract: Hospital Names

It is not necessary to include the names of the hospitals in the abstract. Please remove them from the abstract.

Response:

We agree. The three hospital names have been removed from the Abstract Methods sentence. The sites are now described collectively as three King Saud University-affiliated teaching hospitals in Riyadh, Saudi Arabia, which preserves the geographic and institutional context without listing individual names.

Change in manuscript:

Abstract, Methods sentence: removed '(King Saud University Medical City, King Khalid University Hospital, and King Abdulaziz University Hospital)'. Now reads: 'three King Saud University-affiliated teaching hospitals in Riyadh, Saudi Arabia'.

Comment 6.2 - Abstract: Response Rate Calculation

The response rate calculation requires revision. It should be clearly based on the appropriate sampling frame rather than the total population. Please recheck the calculation and ensure consistency between the sample size determination and the reported response rate.

Response:

We agree the denominator was inappropriate. The previous figure of 58% divided final participants (145) by total enrolled students (250), not by the number approached. The 175 students simultaneously on clinical placement at the three hospitals during the study period constitute the correct sampling frame (based on institutional scheduling records), giving a response rate of 82.9% (145/175). This figure is now used consistently across the Abstract, Methods, and Results.

Change in manuscript:

Abstract Results: 'Of 250 eligible students, 145 completed the questionnaire (response rate = 58%)' corrected to 'Of 175 students approached and meeting eligibility criteria, 145 returned valid questionnaires (response rate = 82.9%)'. Same correction applied in Methods (Sample Size paragraph) and Results (opening sentence).

Comment 6.3 - Background: Missing Citation (Effective Supervision)

Please add a supporting citation for the following statement: 'Effective supervision creates environments where students develop critical thinking, clinical decision-making, and hands-on nursing skills.'

Response:

A citation has been added. We cite Javornicka et al. [3], Zhang et al. [5], and Laugaland et al. [46], all of which directly document associations between supervisory quality and development of clinical reasoning and practical nursing skills.

Change in manuscript:

Background, paragraph 1: Added '[3, 5, 46]' after 'hands-on nursing skills.' Reference [46] (Laugaland et al., BMC Nurs., 2021) added to reference list.

Comment 6.4 - Background: Missing Citations (Supervision Challenges)

Provide appropriate citations for the following statements: 'Despite broad consensus on the value of supervision, significant challenges persist. Clinical instructors often face high workloads and variable caseloads that limit individualised student contact. Supervision quality varies considerably across sites owing to differences in instructor experience, institutional resources, and organisational culture.'

Response:

Citations have been added to each claim. Workload and caseload constraints are now cited with Stevenson et al. [47] and Jokelainen et al. [48]. Site-level variation in supervision quality is cited with Hilli et al. [49] and Chuan and Barnett [50]. All four references have been added to the reference list.

Change in manuscript:

Background, paragraph 2: '[47, 48]' inserted after 'individualised student contact'; '[49, 50]' inserted after 'organisational culture'. References [47]-[50] added to reference list.

Comment 6.5 - Background: Self-Efficacy Mechanism Statement

The authors need to clarify the following statement by specifying the mechanisms and adding appropriate citations: 'In the clinical education context, these mechanisms are operationalised through supervisor feedback (verbal persuasion), direct observation of skilled practitioners (vicarious learning), and graduated practice under supportive conditions (mastery experience).'

Response:

The sentence has been substantially revised. Bandura's four sources of self-efficacy are now named explicitly and in the order he presents them, each linked to a specific clinical education activity, with the applied nursing literature cited alongside Bandura [19]. The revision cites Chesser-Smyth [51], whose phenomenological study directly maps Bandura's mechanisms to student nurse clinical placement experiences.

Change in manuscript:

Background, paragraph 4: Original sentence replaced with: 'In the clinical education context, Bandura's [19] four sources of self-efficacy are operationalised as follows: mastery experience, through supervised clinical tasks of progressively increasing complexity; vicarious learning, through observation of competent instructors and peers performing clinical skills; verbal persuasion, through constructive, specific feedback delivered by supervisors; and management of physiological states, through strategies that reduce anxiety during clinical performance [15, 51].' Reference [51] (Chesser-Smyth, 2005) added to reference list.

Comment 6.6 - Methods: Sample Size and Response Rate Consistency

The sample size is reported as 103 based on calculation and later increased to 145 participants. However, the reported response rate of 58% requires clarification and revision. Please ensure consistency between the sample size calculation, population size, and final included participants.

Response:

This has been clarified in the Methods section. The minimum required sample (n = 103) was derived from the power analysis. The recruitment target was set at 175 - the number of eligible students simultaneously on clinical placement at the three sites - to ensure precision beyond the minimum. Of these, 145 returned valid questionnaires (response rate = 82.9%). The manuscript now clearly distinguishes: (a) power-analysis minimum (103); (b) recruitment target/sampling frame (175); (c) final sample (145; response rate 82.9%).

Change in manuscript:

Methods, Sample Size paragraph: 'To account for an estimated 10-15% non-response rate, the recruitment target was set at 145 participants...' replaced with explanation of 175-student sampling frame, 145 completions, and 82.9% response rate, with institutional source cited.

Comment 6.7 - Methods: Population Size Must Be Referenced

The total population size (250 students) must be properly referenced.

Response:

The figure is now attributed to institutional scheduling records (King Saud University College of Nursing, personal communication, 2024), cited in the revised Methods paragraph in accordance with PLOS ONE reference guidelines for personal communications.

Change in manuscript:

Methods, Sample Size paragraph: Institutional source for the 175-student sampling frame explicitly cited as 'King Saud University College of Nursing, personal communication, 2024'.

Comment 6.8 - Methods: Remove Non-Response Bias Paragraph

I suggest you remove the attached paragraph from the methods section: 'Response rate and non-response bias. Of approximately 250 students enrolled... suggesting the final sample was reasonably representative of the broader population.'

Response:

This paragraph has been deleted in full. As the reviewer correctly notes, because a purposive sampling frame was used, comparing respondents to the total enrolled population is methodologically inconsistent with the design. The deletion is marked as a tracked change in the manuscript.

Change in manuscript:

Methods, Data Collection Procedure section: Paragraph headed 'Response rate and non-response bias' (including chi-square comparisons) deleted in full.

Comment 6.9 - Methods: Revise Response Rate Sentence

The following statement should be revised for clarity and accuracy: 'Of approximately 250 students enrolled in clinical placements at the three hospitals during the study period, 145 returned valid questionnaires (response rate = 58%).'

Response:

This sentence has been deleted as part of the removal of the non-response bias paragraph (Comment 6.8). The corrected response rate (82.9%) now appears in the revised Sample Size paragraph and in the opening sentence of the Results.

Change in manuscript:

See Comments 6.6 and 6.8. Results opening sentence now reads: 'Of 175 students approached and meeting eligibility criteria, 145 returned valid questionnaires (response rate = 82.9%).

Comment 6.10 - Results: Inappropriate ANOVA for Gender in Table 9

In Table 9, ANOVA p-values are reported for variables where ANOVA is not appropriate for several variables such as gender. Please reanalyse the data using appropriate statistical tests.

Response:

We thank the reviewer for identifying this error. For the dichotomous variable gender (male vs. female), ANOVA has been replaced with an independent samples t-test. For variables with three or more categories (year of study; clinical placement location), one-way ANOVA with post-hoc Tukey tests remains appropriate and is retained. The Methods (Statistical Analysis), Table 9 caption, the Table 9 column heading, the Table 9 footnote, and the Results text for Table 9 have all been updated accordingly. The gender p-value in Table 9 now reflects the t-test result (p = 0.318).

Change in manuscript:

Methods: New sentence specifying t-test for dichotomous variables and ANOVA for 3+ category variables. Table 9 caption: 'ANOVA Results' -> 'Statistical Test Results'. Column header: 'p-value (ANOVA)' -> 'p-value (statistical test)'. Gender p-value: 0.373 -> 0.318 with dagger footnote: 'Independent samples t-test used for gender (dichotomous variable); one-way ANOVA used for all other comparisons.' Results text updated to specify test type for each variable.

Comment 6.11 - Results: Response Rate in Opening Sentence

The authors should write the accurate response rate in the first sentence in the results section.

Response:

The opening of the Results has been revised. The cross-reference 'see Methods and and Appendix B' (which also contained a typographic double-word error) has been corrected to 'see Appendix B', and a new sentence has been inserted: 'Of 175 students approached and meeting eligibility criteria, 145 returned valid questionnaires (response rate = 82.9%).'

Change in manuscript:

Results, opening paragraph: Added sentence with corrected response rate (82.9%). Typographic error 'and and' corrected to 'and'.

Comment 6.12 - Results: Table References at End of Paragraphs

The authors must write the number of several tables at the end of the paragraphs, not in the first sentence, such as 'Table 1 presents the sociodemographic characteristics of the sample (n = 145).'

Response:

All Results paragraphs have been reviewed. Where a table number appeared as the grammatical subject of the opening sentence, it has been moved to a parenthetical citation at the end of the paragraph. The example given by the reviewer (Table 1) has been corrected, and equivalent instances elsewhere have been revised.

Change in manuscript:

Results, Sociodemographic Characteristics paragraph: 'Table 1 presents the sociodemographic characteristics of the sample (n = 145). The majority...' -> 'The majority... (Table 1).' Equivalent restructuring applied throughout Results section.

Comment 6.13 - Discussion: Substantial Revision Required

This section requires substantial revision, as its current quality is insufficient for publication standards. The discussion should be rewritten in a more coherent, critical, and evidence-based academic style.

Response:

The Discussion has been substantially revised throughout. Each subsection now: (1) opens with the empirical finding; (2) compares it explicitly with a named prior study using specific values where available; and (3) closes with a theoretically grounded interpretation tied to social cognitive theory. New comparative literature has been incorporated across all subsections (see Comment 6.15 for detail).

Change in manuscript:

Discussion, all subsections: New comparative sentences inserted citing Javornická et al. [3], Woo and Li [13], Phillips et al. [14], Khari et al. [15], Abuadas [20], Donough and Van der Heever [21], Zhang et al. [5], Martin et al. [4], Burgess et al. [39], and Laugaland et al. [2].

Comment 6.14 - Discussion: Avoid Overemphasis on Numerical Values

Avoid overemphasis on numerical values within the discussion section such as 'The significant variation in satisfaction across the three hospitals (ANOVA: F = 4.312, p = 0.016; eta-squared = 0.058) warrants particular attention'.

Response:

This sentence has been revised to: 'Satisfaction differed significantly across the three hospital sites, with a small-to-medium effect size.' The F-value, p-value, and effect size have been removed from the Discussion prose. We applied this principle consistently: all F-values, beta coefficients, and exact p-values that appeared in the Discussion prose have been converted to plain-language descriptions of significance and direction, with readers directed to the Results tables for full statistics.

Change in manuscript:

Discussion, Institutional and Cultural Context subsection: '...The significant variation in satisfaction across the three hospitals (ANOVA: F = 4.312, p = 0.016; eta-squared = 0.058) warrants particular attention.' -> 'Satisfaction differed significantly across the three hospital sites, with a small-to-medium effect size.'

Comment 6.15 - Discussion: Strengthen Comparison with Prior Studies

Several subsections lack adequate comparison with existing literature. Please strengthen the discussion by: (1) comparing findings with relevant previous studies; (2) providing scientific justification for key results; (3) strengthening theoretical and empirical interpretation.

Response:

Each subsection of the Discussion has been augmented with direct empirical comparisons:

(1) Levels

Attachment Submitted filename: Response_to_Reviewers_auresp_4.docx

# Peer review: Decision Letter 4

**DOI:** 10.1371/journal.pone.0352318.r009

**Authors:** Majed Alamri, Majed Alamri, Majed Alamri, Majed Alamri, Majed Alamri

9 Jun 2026

Exploring the Relationship Between Supervisory Support, Self-Efficacy, and Satisfaction Among Nursing Students in Saudi Arabia

PONE-D-26-01430R4

Dear Dr. Alodhailah,

We’re pleased to inform you that your manuscript has been judged scientifically suitable for publication and will be formally accepted for publication once it meets all outstanding technical requirements.

Within one week, you’ll receive an e-mail detailing the required amendments. When these have been addressed, you’ll receive a formal acceptance letter and your manuscript will be scheduled for publication.

An invoice will be generated when your article is formally accepted. Please note, if your institution has a publishing partnership with PLOS and your article meets the relevant criteria, all or part of your publication costs will be covered. Please make sure your user information is up-to-date by logging into Editorial Manager at Editorial Manager® and clicking the ‘Update My Information' link at the top of the page. For questions related to billing, please contact billing support.

If your institution or institutions have a press office, please notify them about your upcoming paper to help maximize its impact. If they’ll be preparing press materials, please inform our press team as soon as possible -- no later than 48 hours after receiving the formal acceptance. Your manuscript will remain under strict press embargo until 2 pm Eastern Time on the date of publication. For more information, please contact onepress@plos.org.

Kind regards,

Majed Sulaiman Alamri, PhD

Academic Editor

PLOS One

Additional Editor Comments (optional):

Reviewers' comments:

Reviewer's Responses to Questions

**Comments to the Author**

1. If the authors have adequately addressed your comments raised in a previous round of review and you feel that this manuscript is now acceptable for publication, you may indicate that here to bypass the “Comments to the Author” section, enter your conflict of interest statement in the “Confidential to Editor” section, and submit your "Accept" recommendation.

Reviewer #4: All comments have been addressed

Reviewer #6: All comments have been addressed

**********

2. Is the manuscript technically sound, and do the data support the conclusions?

The manuscript must describe a technically sound piece of scientific research with data that supports the conclusions. Experiments must have been conducted rigorously, with appropriate controls, replication, and sample sizes. The conclusions must be drawn appropriately based on the data presented.

Reviewer #4: Yes

Reviewer #6: (No Response)

**********

3. Has the statistical analysis been performed appropriately and rigorously?

Reviewer #4: I Don't Know

Reviewer #6: (No Response)

**********

4. Have the authors made all data underlying the findings in their manuscript fully available?

The PLOS Data policy requires authors to make all data underlying the findings described in their manuscript fully available without restriction, with rare exception (please refer to the Data Availability Statement in the manuscript PDF file). The data should be provided as part of the manuscript or its supporting information, or deposited to a public repository. For example, in addition to summary statistics, the data points behind means, medians and variance measures should be available. If there are restrictions on publicly sharing data—e.g. participant privacy or use of data from a third party—those must be specified.

Reviewer #4: Yes

Reviewer #6: (No Response)

**********

5. Is the manuscript presented in an intelligible fashion and written in standard English?

PLOS ONE does not copyedit accepted manuscripts, so the language in submitted articles must be clear, correct, and unambiguous. Any typographical or grammatical errors should be corrected at revision, so please note any specific errors here.

Reviewer #4: Yes

Reviewer #6: (No Response)

**********

6. Review Comments to the Author

Please use the space provided to explain your answers to the questions above. You may also include additional comments for the author, including concerns about dual publication, research ethics, or publication ethics. (Please upload your review as an attachment if it exceeds 20,000 characters)

Reviewer #4: Thank you for addressing all the comments in my previous reviews and the comments from all reviewers. I am now satisfied with the current version of the manuscript.

Reviewer #6: (No Response)

**********

7. PLOS authors have the option to publish the peer review history of their article (what does this mean?). If published, this will include your full peer review and any attached files.

If you choose “no”, your identity will remain anonymous but your review may still be made public.

**Do you want your identity to be public for this peer review?**  For information about this choice, including consent withdrawal, please see our Privacy Policy.

Reviewer #4: No

Reviewer #6: No

**********

# Editor report: Acceptance letter

**DOI:** 10.1371/journal.pone.0352318.r010

**Authors:** Majed Alamri, Majed Alamri, Majed Alamri, Majed Alamri, Majed Alamri

PONE-D-26-01430R4

PLOS One

Dear Dr. Alodhailah,

I'm pleased to inform you that your manuscript has been deemed suitable for publication in PLOS One. Congratulations! Your manuscript is now being handed over to our production team.

At this stage, our production department will prepare your paper for publication. This includes ensuring the following:

* All references, tables, and figures are properly cited

* All relevant supporting information is included in the manuscript submission,

* There are no issues that prevent the paper from being properly typeset

You will receive further instructions from the production team, including instructions on how to review your proof when it is ready. Please keep in mind that we are working through a large volume of accepted articles, so please give us a few days to review your paper and let you know the next and final steps.

Lastly, if your institution or institutions have a press office, please let them know about your upcoming paper now to help maximize its impact. If they'll be preparing press materials, please inform our press team within the next 48 hours. Your manuscript will remain under strict press embargo until 2 pm Eastern Time on the date of publication. For more information, please contact onepress@plos.org.

You will receive an invoice from PLOS for your publication fee after your manuscript has reached the completed accept phase. If you receive an email requesting payment before acceptance or for any other service, this may be a phishing scheme. Learn how to identify phishing emails and protect your accounts at https://explore.plos.org/phishing.

If we can help with anything else, please email us at customercare@plos.org.

Thank you for submitting your work to PLOS ONE and supporting open access.

Kind regards,

PLOS ONE Editorial Office Staff

on behalf of

Prof. Majed Sulaiman Alamri

Academic Editor

PLOS One
